# tRNA biogenesis and specific aminoacyl-tRNA synthetases regulate senescence stability under the control of mTOR

**DOI:** 10.1371/journal.pgen.1009953

**Published:** 2021-12-20

**Authors:** Jordan Guillon, Hugo Coquelet, Géraldine Leman, Bertrand Toutain, Coralie Petit, Cécile Henry, Alice Boissard, Catherine Guette, Olivier Coqueret

**Affiliations:** Centre de Recherche en Cancérologie et Immunologie Nantes Angers (CRCINA), INSERM U1232 Paul Papin ICO Cancer Center, Université d’Angers, Angers, France; New York Medical College Graduate School of Basic Medical Sciences, UNITED STATES

## Abstract

Oncogenes or chemotherapy treatments trigger the induction of suppressive pathways such as apoptosis or senescence. Senescence was initially defined as a definitive arrest of cell proliferation but recent results have shown that this mechanism is also associated with cancer progression and chemotherapy resistance. Senescence is therefore much more heterogeneous than initially thought. How this response varies is not really understood, it has been proposed that its outcome relies on the secretome of senescent cells and on the maintenance of their epigenetic marks. Using experimental models of senescence escape, we now described that the stability of this proliferative arrest relies on specific tRNAs and aminoacyl-tRNA synthetases. Following chemotherapy treatment, the DNA binding of the type III RNA polymerase was reduced to prevent tRNA transcription and induce a complete cell cycle arrest. By contrast, during senescence escape, specific tRNAs such as tRNA-Leu-CAA and tRNA-Tyr-GTA were up-regulated. Reducing tRNA transcription appears necessary to control the strength of senescence since RNA pol III inhibition through BRF1 depletion maintained senescence and blocked the generation of escaping cells. mTOR inhibition also prevented chemotherapy-induced senescence escape in association with a reduction of tRNA-Leu-CAA and tRNA-Tyr-GTA expression. Further confirming the role of the tRNA-Leu-CAA and tRNA-Tyr-GTA, results showed that their corresponding tRNA ligases, LARS and YARS, were necessary for senescence escape. This effect was specific since the CARS ligase had no effect on persistence. By contrast, the down-regulation of LARS and YARS reduced the emergence of persistent cells and this was associated with the modulation of E2F1 target genes expression. Overall, these findings highlight a new regulation of tRNA biology during senescence and suggest that specific tRNAs and ligases contribute to the strength and heterogeneity of this tumor suppressive pathway.

## Author summary

Senescence is a tumor suppressive mechanism induced in response to oncogenes or chemotherapy. Senescence was initially defined as a definitive arrest of cell proliferation but doubts have emerged as to the value of this mechanism in terms of suppression. Recent findings published by several laboratories including our own have shown that some cells escape senescence to become more transformed. This study shows that different tRNAs are expressed in growing, senescent or emerging cells. The tRNA-Leu-CAA and tRNA-Tyr-GTA are up-regulated during senescence escape whereas this was not the case of the other tRNAs tested. In addition, using proteomic analysis and inactivation experiments, we found that the corresponding tRNA ligases, YARS for tRNA-Tyr-GTA and LARS for the tRNA-Leu-CAA, are necessary for senescence escape. Results also show that the expression of the tRNA-Leu-CAA and tRNA-Tyr-GTA are controlled by the mTOR pathway and that this kinase is necessary for senescence escape. Reducing tRNA transcription appears necessary to control the strength of senescence since RNA pol III inhibition maintained senescence and blocked the generation of escaping cells. In light of these results, we propose the hypothesis that the heterogeneity of tRNAs and ligases expression leads to distinct states of light or deep senescence.

## Introduction

Senescence induces a definitive proliferative arrest in response to telomere shortening, oncogenes or chemotherapy [1,2]. This tumor suppressive mechanism is most of the time induced by DNA damage and activation of the p53-p21 and p16-Rb pathways. A definitive proliferative arrest is then maintained by the Rb-mediated compaction of proliferative genes within heterochromatin foci or SAHFs (Senescence Associated Heterochromatin Foci) [3]. Senescence is also characterized by the production of a specific secretome known as the SASP (Senescence-Associated Secretory Phenotype). Mainly composed of cytokines and chemokines, the SASP maintains senescence and attracts immune cells which then eliminate the senescent populations [4–7]. Thus, through the up-regulation of the p53 and Rb pathways and the activation of immune responses, senescence prevents the propagation of abnormal cells.

Although initially described as a definitive proliferative arrest, we and others have recently shown that some cells can escape senescence, indicating that distinct stages of light and deep senescence should be distinguished [2,7–9]. In particular, we have proposed that this pathway functions as an adaptive mechanism in response to chemotherapy-induced senescence (CIS) [7,9]. We have described that cancer cells can escape CIS and emerge as more transformed cells that resist anoïkis and are more invasive [10–13]. Cells that present an incomplete senescence response are characterized by a reduced expression of CD47 [13]. As previously proposed [14,15], this indicates that senescent populations are heterogeneous. This heterogeneity can be explained by a variable expression of p16 which is necessary for senescence maintenance [16]. Several studies have also reported that the composition of the SASP varies and that this secretome can also enhance tumor progression [17–19]. In this context, the mTOR (mammalian target of rapamycin) kinase controls the expression of the SASP and the tumor-promoting activity of senescent cells, through the regulation of IL-1 alpha translation and of the RNA-binding protein ZFP36L1 [20,21].

Taken together, these studies indicate that senescence is much more dynamic than initially thought and that a better characterization of these arrested cells is necessary. In this study, we pursued our experiments with the aim of characterizing the signaling pathways involved in the maintenance of senescence. During the initial steps of this suppressive arrest, we describe that the transcription of the type III RNA polymerase is down-regulated and that tRNA synthesis is then reactivated during senescence escape. Depending on the experimental model, results showed that the expression of specific tRNAs was reactivated, such as the tRNA^Leu-^CAA and tRNA^Tyr-^GTA in emergent colorectal and breast cells or breast cancer organoids. In addition, specific aminoacyl-tRNA synthetases (ARS) such as the Leucyl-and Tyrosyl-tRNA ligases were necessary for senescence escape. Our results also indicate that this deregulation of tRNA synthesis led to the activation of the Unfolded Protein Response (UPR) and that this tRNA-mediated ER stress is resolved by mTOR to allow senescence escape. These observations extend previous results showing that tRNAs have unexpected functions during cancer progression [22,23]. In particular, it has been recently reported that the tRNA^Glu^ and tRNA^Arg^ are over-expressed in metastatic cells and that their inactivation reduces the *in vivo* metastasis of breast cancer cells [24].

Altogether, these results indicate that specific pools of tRNAs or ARSs regulate the outcome of this suppressive response and of chemotherapy. We propose that different types of senescence, replicative, oncogenic or mediated by chemotherapy might lead to the expression of different pools of tRNA and ARSs. This could partly explain the variability of the senescence and the deleterious effects of this pathway in response to treatment.

## Results

### mTOR activity is necessary for CIS escape

**Fig 1:**
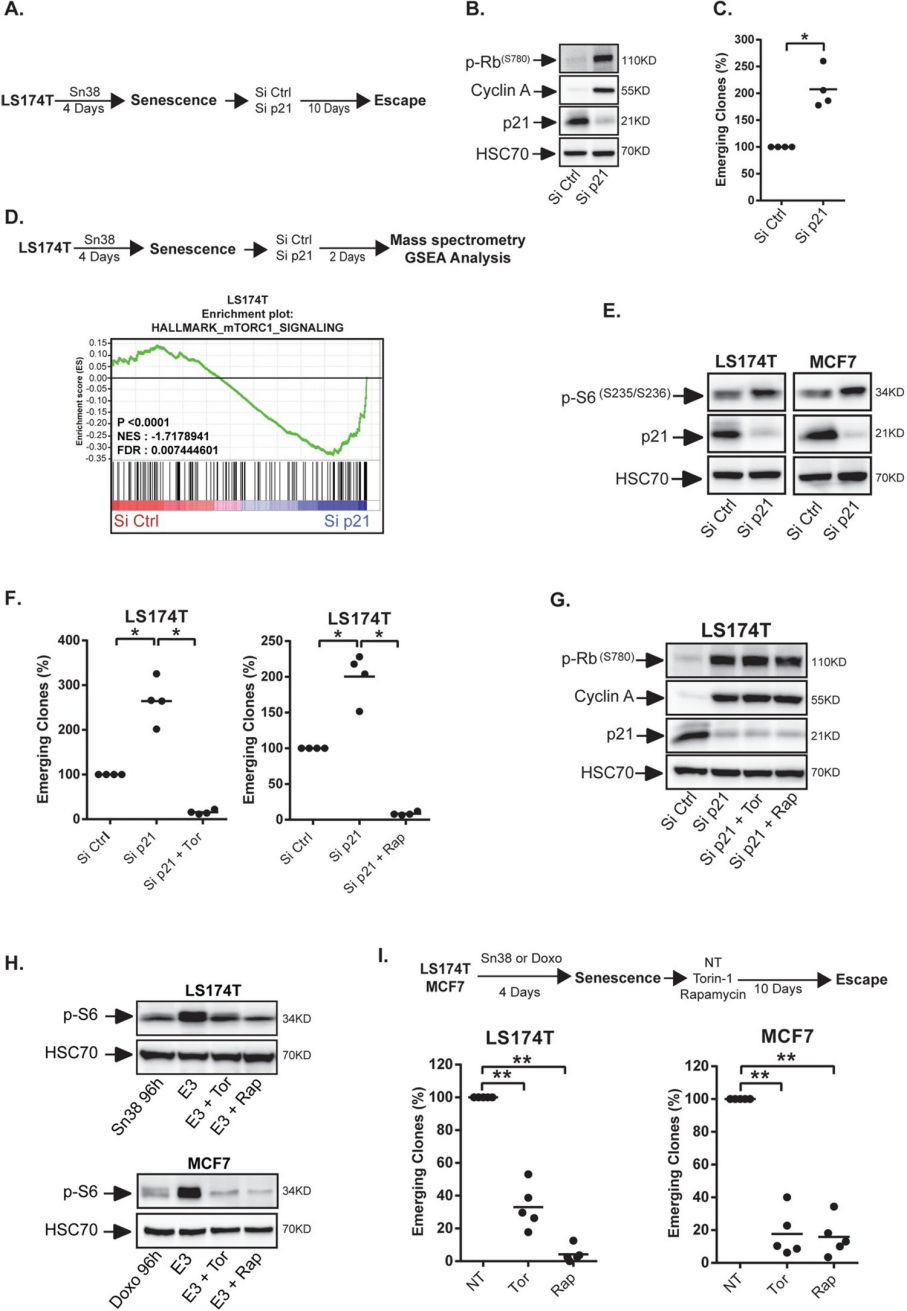
mTOR is necessary for senescence escape. A. Senescence was induced by treating LS174T colorectal cells with the topoisomerase I inhibitor sn38 as indicated. Cells were then washed with PBS and transfected with a control siRNA or a siRNA directed against p21 for 24 hr and senescence escape was generated by adding 10% FBS. B. LS174T cells were treated as above and cell extracts were recovered 2 days after p21 depletion. The expression of the indicated proteins was analyzed by western blot (n = 3). C. Number of emerging clones analyzed after p21 inactivation (n = 4, Kolmogorov-Smirnov test * = p<0.05). D. Senescent cells were transfected with a control siRNA or a siRNA directed against p21. Cell extracts were analyzed by SWATH quantitative proteomics and GSEA analysis 2 days after p21 depletion (n = 3). E. Validation of mTORCI activation by western blot 2 days after p21 inactivation in LS174T and MCF7 mammary cells (n = 3). F and G. Senescent LS174T cells were transfected with a control siRNA or a siRNA directed against p21 for 24 hr. Cells were then stimulated with 10% FBS in the presence or absence of mTOR inhibitors (Rapamycin: 5nM, Torin-1: 15nM). The number of emerging clones was evaluated after 10 days (F, n = 4, Kolmogorov-Smirnov test * = p<0.05). Cell extracts were recovered after 2 days and the expression of the indicated proteins was analyzed by western blot (G, n = 3). H. Senescent cells were generated as above and emergence was induced by adding 10% FBS. mTORCI activation was analyzed by western blot 3 days after the addition of serum on senescent cells (noted E3 on the figure, n = 3 for MCF7, 2 for LS174T). I. Following senescence induction, cells were stimulated with 10% FBS in the presence or absence of mTOR inhibitors. The number of emerging clones was evaluated 10 days later (n = 5, Kolmogorov-Smirnov test ** = p<0.01).

We have previously shown that LS174T colorectal cells and MCF7 breast cells enter senescence when treated respectively with sn38 or doxorubicin [10–13]. Both drugs are commonly used in first-line chemotherapy treatments; sn38, a topoisomerase I inhibitor, is the active metabolite of irinotecan which is one of the main treatment of colorectal cancer. CIS induction was confirmed in this study by showing the up-regulation of p21, SA-β-galactosidase staining and SASP production in the two cell lines (S1 Fig, note that control cells are always treated with the DMSO vehicle). In these breast and colorectal cell lines that do not express p16INK4, we have previously reported that p21 maintains senescence and that its down-regulation allows CIS escape [13]. To confirm this observation, senescence was induced with sn38 for 96hr, cells were then transfected with control siRNA or siRNA directed against p21 and the number of re-proliferating cells was evaluated after 10 days by crystal violet staining (Fig 1A, re-proliferating cells are described as emerging clones in the figures). Western blot analysis confirmed the down-regulation of the cell cycle inhibitor and showed a concomitant up-regulation of cyclin A and of phospho-Rb (Fig 1B). As we have previously shown [13], p21 inactivation led to a significant increase in CIS escape (Fig 1C). This effect was observed in LS174T colorectal cells and in MCF7 breast cells (S2A Fig). We then used mass spectrometry to analyze the signaling pathways involved in CIS escape. To this end, p21 was inactivated in senescent cells, extracts were collected after 48hr and analyzed by SWATH-MS approaches as we recently described [25]. GSEA analysis indicated that Myc and E2Fs proliferative pathways were reactivated as expected (see S9 Fig). Interestingly, a significant up-regulation of mTOR signaling was also detected (Fig 1D). We focused on this kinase since it plays a key role during senescence [20,21,26–28]. Western blot analysis confirmed that mTOR was activated in LS174T and MCF7 cells following p21 inhibition, as shown by the phosphorylation of the ribosomal S6 and 4E-BP1 proteins (Figs 1E and S2B). We then asked if mTOR was involved in CIS escape by treating the cells at the beginning of emergence with torin-1 and rapamycin, two common drugs used to inactivate this kinase. These inhibitors significantly blocked CIS escape despite p21 inactivation (Fig 1F). We chose to used torin-1 and rapamycin at low concentrations (Rapamycin (5 nM) and Torin-1 (15nM)). It should be noted that under these conditions, mTOR inhibition also reduced the proliferation of non-treated LS174T and MCF7 cells (S2D Fig). Note that at these concentrations, the effect of torin-1 and rapamycin was less significant in non-treated cells as compared to its effect during senescence escape. In addition, Rb phosphorylation or cyclin A expression were not inhibited by the two drugs (Fig 1G). Finally, to confirm this observation, we down-regulated p21 and Raptor, a mTOR binding protein essential for its activity. Results presented S2C Fig show that Raptor inhibition reduced senescence escape following p21 inhibition.

We then determined if mTOR was also involved in spontaneous CIS escape, in the absence of p21 manipulation. Western blot analysis indicated that this pathway was activated at the early steps of emergence as shown by the phosphorylation of the ribosomal S6 and ULK1 proteins (Figs 1H and S2E). In this condition, Torin-1 or rapamycin inhibited its activity and significantly reduced CIS escape, both in LS174T and MCF7 cells (Fig 1H and 1I). The same effect was observed when Raptor was down-regulated by siRNA (S2F Fig). In light of these results, we determined if mTOR inhibition led to an enhanced senescence stability. To this end, we used ß-galactosidase staining to determine if mTOR inhibition increased the number of senescent cells. In control conditions, we observed after 7 days the emergence of dividing, white cells, in the middle of blue populations that remain senescent (S3A Fig). This was not the case when the kinase was inhibited, suggesting that the senescence arrest might be more stable. We have recently shown that senescent cells can be identified by an increased expression of the CD47 receptor [13]. On the opposite, the dividing, emergent clones have a reduced expression of the receptor and can be identified as CD47^low^ cells. Using FACS experiments, we analyzed CD47 expression during emergence, in the presence or absence of Torin-1 or rapamycin. Interestingly, we found that mTOR inhibition increased the proportion of CD47^high^ cells which correspond to the population that remain senescent (S3B and S3C Fig).

Altogether, these results indicate that mTOR inhibition increased the stability of chemotherapy-mediated senescence, both in LS174T and MCF7 cells.

### mTOR promotes CIS escape under protein stress conditions

**Fig 2:**
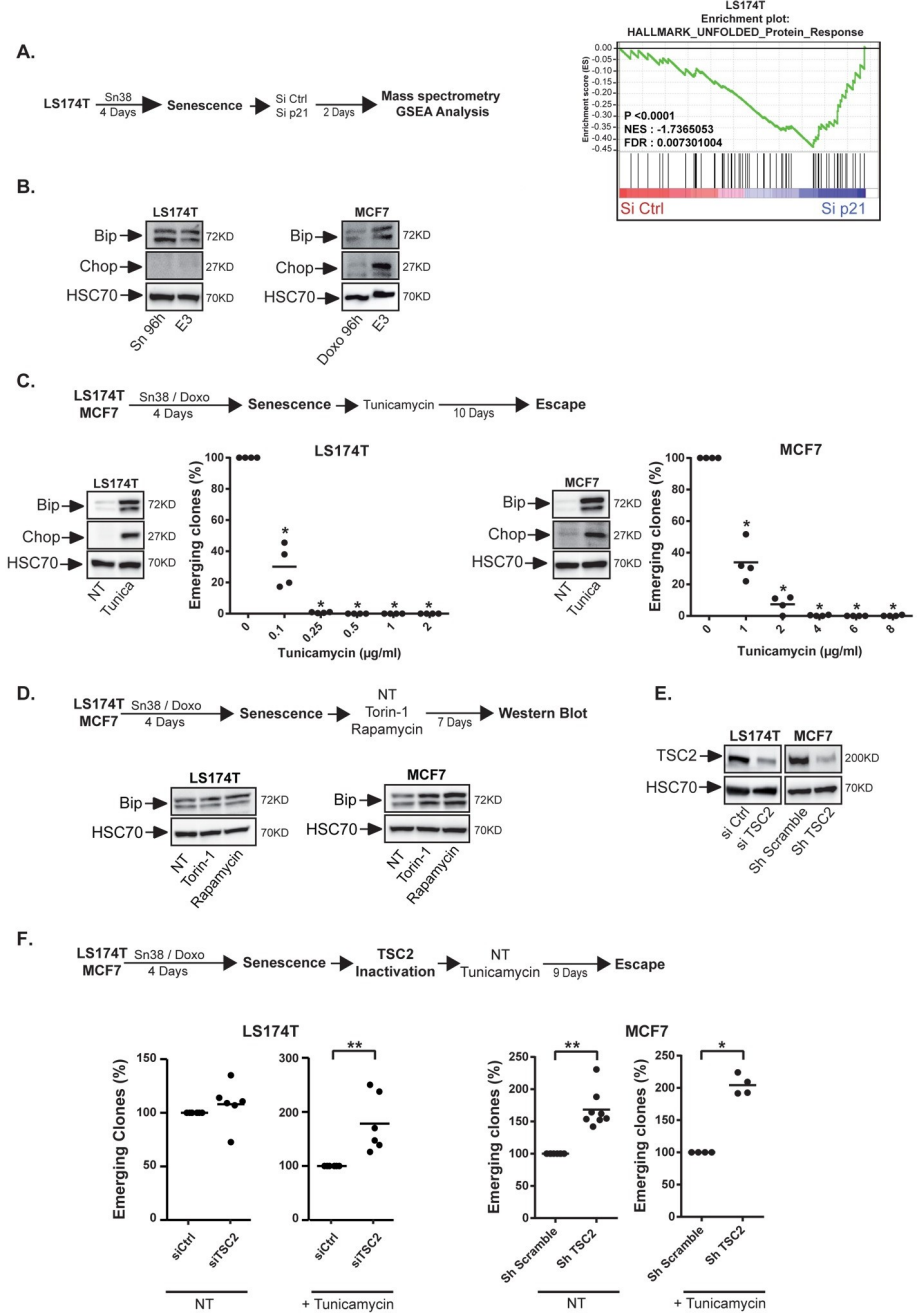
mTOR allows senescence escape in the presence of an ER stress. A. Senescent cells were transfected with a control siRNA or a siRNA directed against p21. Cell extracts were analyzed by SWATH quantitative proteomics and GSEA analysis 2 days after p21 depletion (n = 3). B. Analysis of the expression of the UPR markers in LS174T and MCF7 senescent cells after 3 days of emergence (noted E3 on the figure, n = 3). C. Senescent MCF7 and LS174T cells were treated with increasing concentrations of tunicamycin as indicated and the number of emerging clones was evaluated 10 days later (n = 4, Kolmogorov-Smirnov test, * = p<0.05). UPR induction was validated by Western blot 24 hr after the treatment of senescent cells with the lowest concentration of tunicamycin (LS174T: 0.1 μg/ml, n = 2; MCF7:1 μg/ml, n = 2). D. After senescence induction, cells were stimulated with 10% FBS in the presence or absence of mTOR inhibitors. Cell extracts were recovered 7 days later and the expression of Bip was analyzed by western blot (n = 3). E, F. Following senescence induction, cells were transduced with a control shRNA or a shRNA directed against TSC2 (MCF7) or with a control siRNA or a siRNA directed against TSC2 (LS174T cells). TSC2 inhibition was validated by western blot 2 days after transduction (E. LS174T n = 2, MCF7 n = 1). F: Cells were then washed and treated or not with tunicamycin (LS174T: 0.1μg/ml, MCF7 1μg/ml) and emergence was then evaluated as above (n = 4 to 8, Kolmogorov-Smirnov test, * = p<0.05 ** = p<0.01).

The mass spectrometry analysis also detected a significant deregulation of the Unfolded Protein Response (UPR) when cell emergence was induced by p21 inactivation (Fig 2A). This was confirmed during spontaneous CIS escape, as evidenced by an increased expression of Bip and Chop, two main sensors of ER stress signaling. Note that this was detected in emergent MCF7 cells but not in LS174T cells, suggesting that the two cell lines resolved this stress differently (Fig 2B). This suggested that cell emergence might be associated with the ability to resolve this protein stress. To test this hypothesis, we used tunicamycin or thapsigargin, two well-known inducers of the UPR pathway. When added on senescent cells, these two drugs significantly blocked CIS escape. Bip and Chop expressions were increased as expected (Figs 2C and S4A). Since mTOR inhibition prevented cell emergence, we then asked if this was related to an abnormal activation of this stress pathway. A significant increase of Bip expression was detected during emergence when MCF7 senescent cells were treated with Torin-1 or rapamycin (Fig 2D). This effect was less evident in LS174T cells, again suggesting that the colorectal cell line might resolve the protein stress more efficiently. To extend this observation, we activated mTOR using a lentivirus encoding an shRNA directed against TSC2. As an inhibitor of the Rheb GTPase, TSC2 is one of the main repressors of the mTOR kinase [29]. MCF7 cells were infected after CIS induction, and senescent cells were treated with tunicamycin to increase the UPR stress and block emergence as described Fig 2C. TSC2 expression was reduced and the mTOR signaling pathway was activated as expected (Figs 2E and S4B). Results showed that mTOR activation allowed CIS escape despite the increased proteotoxic stress generated by the drug (right part of Fig 2F). To confirm this observation, TSC2 was inactivated in LS174T cells with a different siRNA sequence (Figs 2E and S4C). Results showed that mTOR activation also allowed CIS escape despite the increased protein stress generated in the colorectal cell line (left part of Fig 2F).

Together, these results indicate that a high UPR stress prevents CIS escape and that mTOR promotes cell emergence when this stress is increased.

### mTOR Regulates tRNA biogenesis during CIS escape

**Fig 3:**
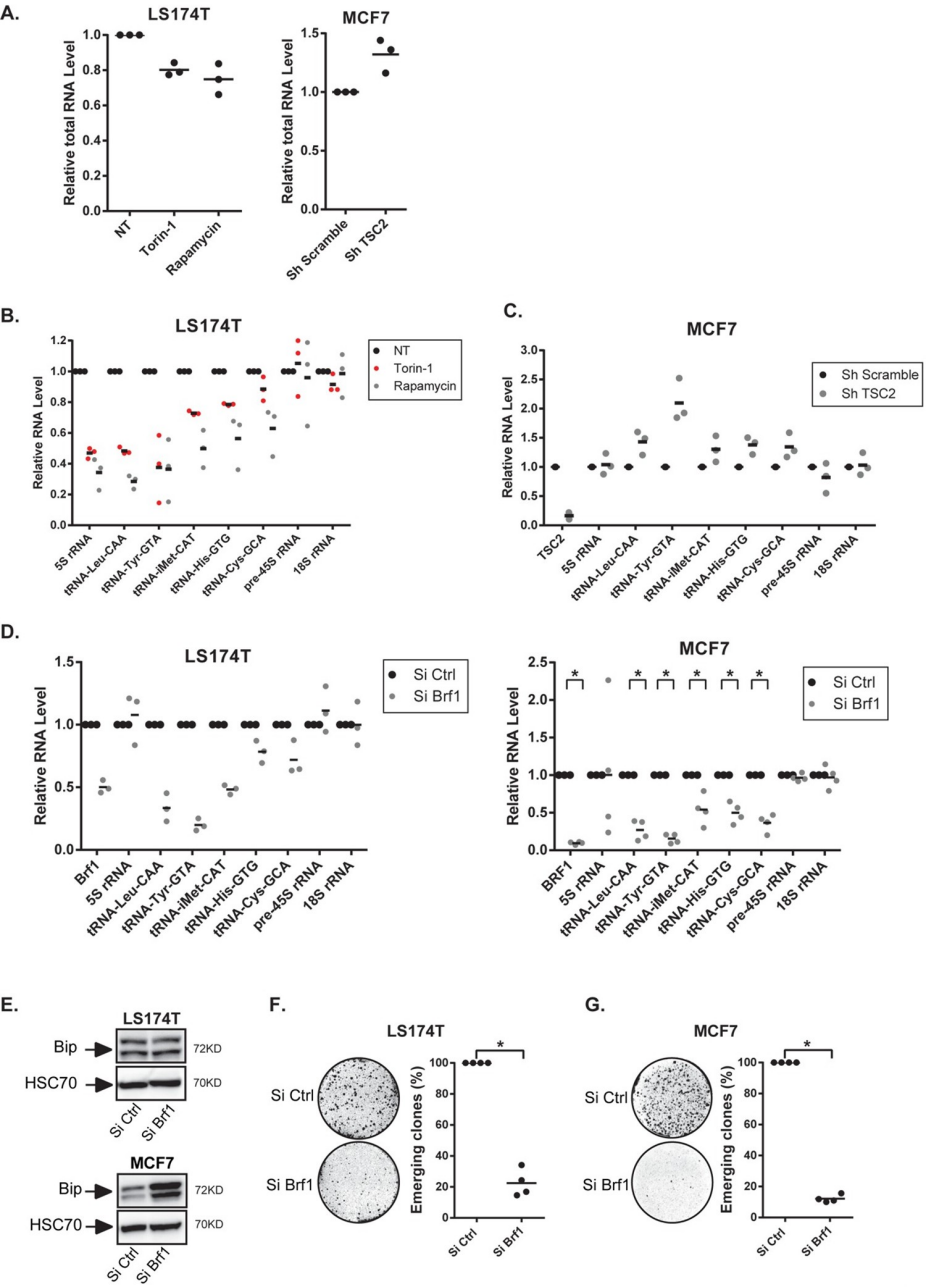
mTOR regulates RNA Pol III activity during CIS escape. A. Nanodrop quantification of total RNA level in senescent LS174T cells two days after mTOR inhibition (left, n = 3) or in senescent MCF7 cells two days after TSC2 inhibition (right, n = 3). B. Senescent LS174T cells were treated with mTOR inhibitors, and the relative expression of the indicated RNA was analyzed by RT-QPCR after 7 days (n = 3). C. Analysis of the expression of the indicated RNAs 2 days after TSC2 depletion in MCF7 senescent cells by RT-QPCR (n = 3). D. Senescent LS174T and MCF7 cells were transfected with a control siRNA or a siRNA directed against Brf1 during 24 hr and then washed with PBS and stimulated with fresh media. Cell extracts were recovered 2 days after Brf1 depletion and the expression of the indicated RNAs was analyzed by RT-QPCR (LS174T: n = 3; MCF7: n = 4, Kolmogorov-Smirnov test, * = p<0.05). E. Analysis of Bip expression by western blot 7 days after BRF1 depletion in LS174T and MCF7 senescent cells (n = 3). F, G. Senescent LS174T and MCF7 cells were transfected with a control siRNA or a siRNA directed against Brf1 during 24 hr and then washed with PBS and stimulated with fresh media. Nine days after Brf1 inactivation, the number of emerging clones was analyzed (n = 4, Kolmogorov-Smirnov test, * = p<0.05).

We then tried to understand the link between CIS escape, mTOR and the ER stress. In LS174T cells, we noticed that rapamycin and torin-1 always reduced the total RNA concentration during senescence escape. Conversely, when TSC2 was inactivated in MCF7 cells, this led to an increased RNA concentration (Fig 3A). Since mTOR is a global activator of tRNA transcription in cancer cells [30], we determined if this was also the case during CIS escape. We focused on the tRNA-Leu and tRNA-Tyr which have been shown to be regulated by the mTOR pathway [30]. Results presented Fig 3B indicate that the expression of a panel of several tRNAs was reduced when senescent LS174T cells were treated with Torin-1 or rapamycin. The tRNA^Tyr-^GTA and the tRNA^Leu-^CAA were more significantly down-regulated than the other tRNAs tested. Conversely, when mTOR was activated by the depletion of TSC2 in emergent MCF7 cells, we observed a more significant up-regulation of the tRNA^Tyr-^GTA as compared to the other tested tRNAs (Fig 3C). mTOR inhibition also reduced the expression of the 5S rRNA but did not affect RNA polymerase I targets such as the pre45S and 18S rRNAs.

In light of these results, we then determined if a global inhibition of tRNA synthesis could lead to an increased ER stress. To this end, we inactivated BRF1, the main RNA Pol III activator, in senescent cells. The down-regulation of BRF1 was verified by RT-QPCR (Fig 3D, left) or by Western Blot (S5A Fig, compare lane 1 and 2). As expected, this reduced tRNA expression in MCF7 and LS174T cells (Fig 3D). Note that BRF1 inhibition did not significantly reduce the expression of the 5S RNA in senescent cells. In growing cells, the inhibition of the expression of the 5S RNA was observed as well as the down-regulation of the tRNA^Leu-^CAA and tRNA^Tyr-^GTA tRNAs (S5B Fig). BRF1 inactivation led to a significant induction of the Bip sensor in MCF7 cells (Fig 3E). This was not observed in LS174T cells, again indicating that these cells may tolerate a higher level of protein stress. Moreover, when BRF1 was down-regulated, a significant inhibition of senescence escape was observed in the two cell lines (Fig 3F and 3G). This result was confirmed with a different siRNA (S5A and S5C Fig). After 7 days of emergence and cell fixation, a microscopic examination allowed the visualization of emergent, dividing, white clones in the middle of blue senescent cells identified by SA-ß-galactosidase staining. These dividing, white clones were completely absent when BRF1 was inactivated, indicating that reducing the activity of RNA pol III allowed the maintenance of senescence (S5D Fig).

Altogether, these results indicate that mTOR inhibition reduced tRNA expression and in particular the tRNA^Tyr-^GTA and the tRNA^Leu-^CAA during CIS escape. This led to an increased ER stress indicating that mTOR and RNA Pol III activity are necessary to tolerate the protein stress during cell emergence.

### Regulation of tRNA expression during senescence and cell emergence

**Fig 4:**
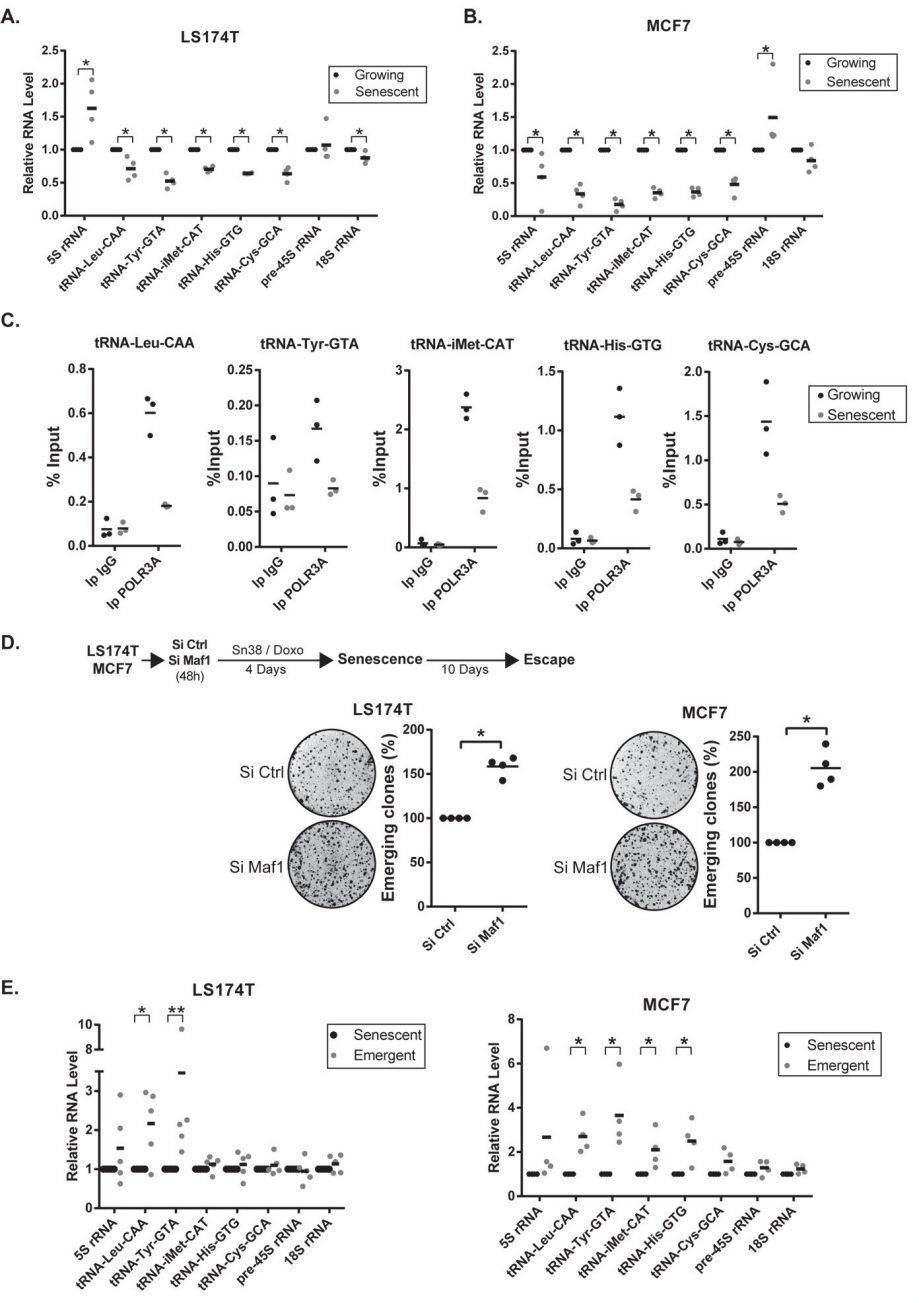
Regulation of tRNA synthesis during senescence and CIS escape. A, B. Expression of the indicated RNAs in growing or senescent cells (LS174T n = 4, MCF7 n = 4, Kolmogorov-Smirnov test, * = p<0.05). C. ChIP analysis of the binding of the type III RNA polymerase (POLR3A) in senescent or growing LS174T cells (n = 3). D. LS174T and MCF7 were transfected with a control siRNA or a siRNA directed against Maf1 48h prior to chemotherapeutic treatment. After senescence induction, cells were washed with PBS and stimulated with fresh media. Ten days later, the number of emerging clones was analyzed (n = 4, Kolmogorov-Smirnov test, * = p<0.05). E. Analysis of the expression of the indicated RNAs in senescent or emergent cells (after 7 days, LS174T n = 5, MCF7, n = 4, Kolmogorov-Smirnov test, * = p<0.05, ** = p<0.01).

These observations suggested that an up-regulation of tRNA pathways could be necessary to promote CIS escape. To test this hypothesis, we first analyzed tRNA expression in growing and senescent cells. RT-QPCR results indicated that tRNA expression was down-regulated in senescent cells, both in LS174T and MCF7 cells (Fig 4A and 4B). In contrast, the expression of the 18S and pre-45S ribosomal RNAs was not significantly modified, further indicating that the activity of RNA pol I was not affected under these experimental conditions. Surprisingly, results also show that the 5S RNA was regulated differently in senescent MCF7 or LS174T cells. Using chromatin immunoprecipitation experiments, we also observed that the type III RNA polymerase was recruited to the tRNA promoters in growing cells but that its binding was significantly reduced in senescent populations (Fig 4C). These observations suggested to us that this reduction of RNA pol III activity might be a necessary step of senescence induction. To test this hypothesis, we inactivated MAF1 in growing cells and then induced senescence in LS174T or MCF7 cells. MAF1 is a repressor of RNA Pol III and its down-regulation enhances tRNA transcription [30–32]. As presented Fig 4D, the down-regulation of MAF1 led to a significant increase of CIS escape. This effect was specific since MAF1 inactivation did not significantly modify the proliferation of non-treated, LS174T growing cells and had a limited effect on MCF7 cells (S6A Fig). Thus, a reduction of RNA Pol III transcription during the early step of senescence is necessary to allow a complete proliferative arrest.

We then determined if tRNA expression was up-regulated during spontaneous CIS escape. A significant increase of the tRNA^Leu-^CAA and tRNA^Tyr-^GTA tRNAs was observed in emergent LS174T cells. The same effect was observed in MCF7 cells where several tRNAs were reactivated (Fig 4E and 4F). Note that all tRNAs were not up-regulated in emergent LS174T cells and that the tRNA^Cys^ was not significantly modified in both cell lines. The expression of the 45S and 18S rRNAs was also not modified. We then determined if this effect was related to senescence or to a consequence of chemotherapy treatment and cell cycle arrest. To this end, we repeated these experiments in MCF7 cells treated with a lower dose of doxorubicin. This lower concentration reduced the number of cells in S phase to the same extent, which was not related to senescence (S6B Fig). In this case, no significant inhibition of tRNA transcription was observed and a slight and general reactivation was observed 3 days after the removal of doxorubicin (S6C and S6D Fig).

**Fig 5:**
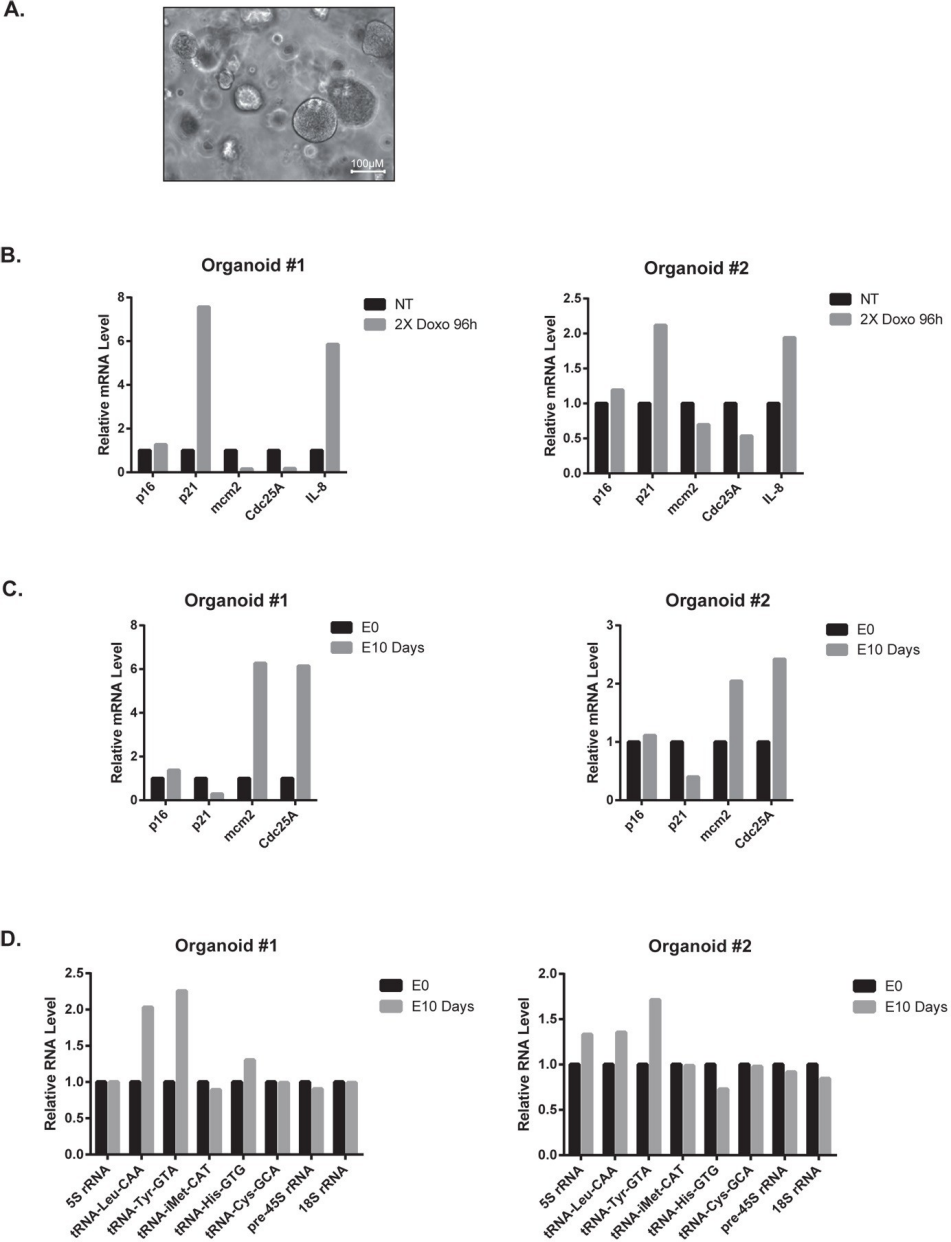
Up-regulation of the tRNA^Leu^-CAA and tRNA^Tyr^-GTA in breast organoids that escape chemotherapy. A. Representative image of breast cancer organoids. B. Analysis by RT-QPCR of proliferative and senescence markers of two breast tumor organoids treated or not with 2 cycles of 96 hr of Doxorubicin (All experiments were performed on two organoids coming from two patients, #1: Doxorubicin 25 ng/ml, #2: Doxorubicin 50ng/ml). C, D. Analysis of the expression of the indicated RNAs on PDOs at the end of the doxorubicin treatment (E0) and 10 days later (E10).

To extend these observations, we then determined if tRNA transcription was also reactivated in patients-derived organoids (PDO, see [33]) that escape chemotherapy (see a representative image Fig 5A). PDOs isolated from two different patients were treated with doxorubicin and experiments indicated that a proliferative arrest was induced after two sequential drug treatments for 96hr. As these cells grew in three dimensions, the SA-ß-galactosidase staining was constitutively positive within the inner part of the spheroid, precluding the use of this marker. RT-QPCR experiments showed that proliferative genes such as MCM2 or CDC25A were down-regulated, together with an up-regulation of IL-8 as an illustrative member of the SASP (Fig 5B). P21 expression was also increased in the two PDOs but this was not the case of p16, probably because this gene was already inactivated. After chemotherapy treatment, some PDOs restarted proliferation and this was illustrated after 10 days by the reactivation of MCM2 and CDC25A and the down-regulation of p21 (Fig 5C). To determine if senescent cells were involved in the generation of escaping cells, we used ABT-263 which is widely recognized as a senolytic compound. We first validated its effect on MCF7 and LS174T cells. Results presented in S7A and S7B Fig show that this drug weakly modified the proliferation of parental cells whereas it completely blocked the senescence escape of sn38 and doxorubicin-treated cells. When used on PDOs, ABT-263 reduced to some extent the viability of untreated spheroids, probably because senescent cells are present within the inner mass of these spheroids (S7C Fig). Interestingly, chemotherapy escape was reduced by the use of ABT-263, indicating that senescent cells present within the PDOs participate in the emergence (S7C Fig). Finally, we evaluated tRNA expression after 10 days of emergence. Results presented in Fig 5D indicate that the tRNA^Leu-^CAA and tRNA^Tyr-^GTA were also up-regulated. This was not the case for the other tRNAs and ribosomal RNAs tested.

Therefore, in two different conditions of treatment escape, we observed an up-regulation of specific tRNAs such as the tRNA^Leu-^CAA and tRNA^Tyr-^GTA when cancer cells resumed proliferation following chemotherapy treatment.

### The LARS and YARS tRNA ligases promote CIS escape

**Fig 6:**
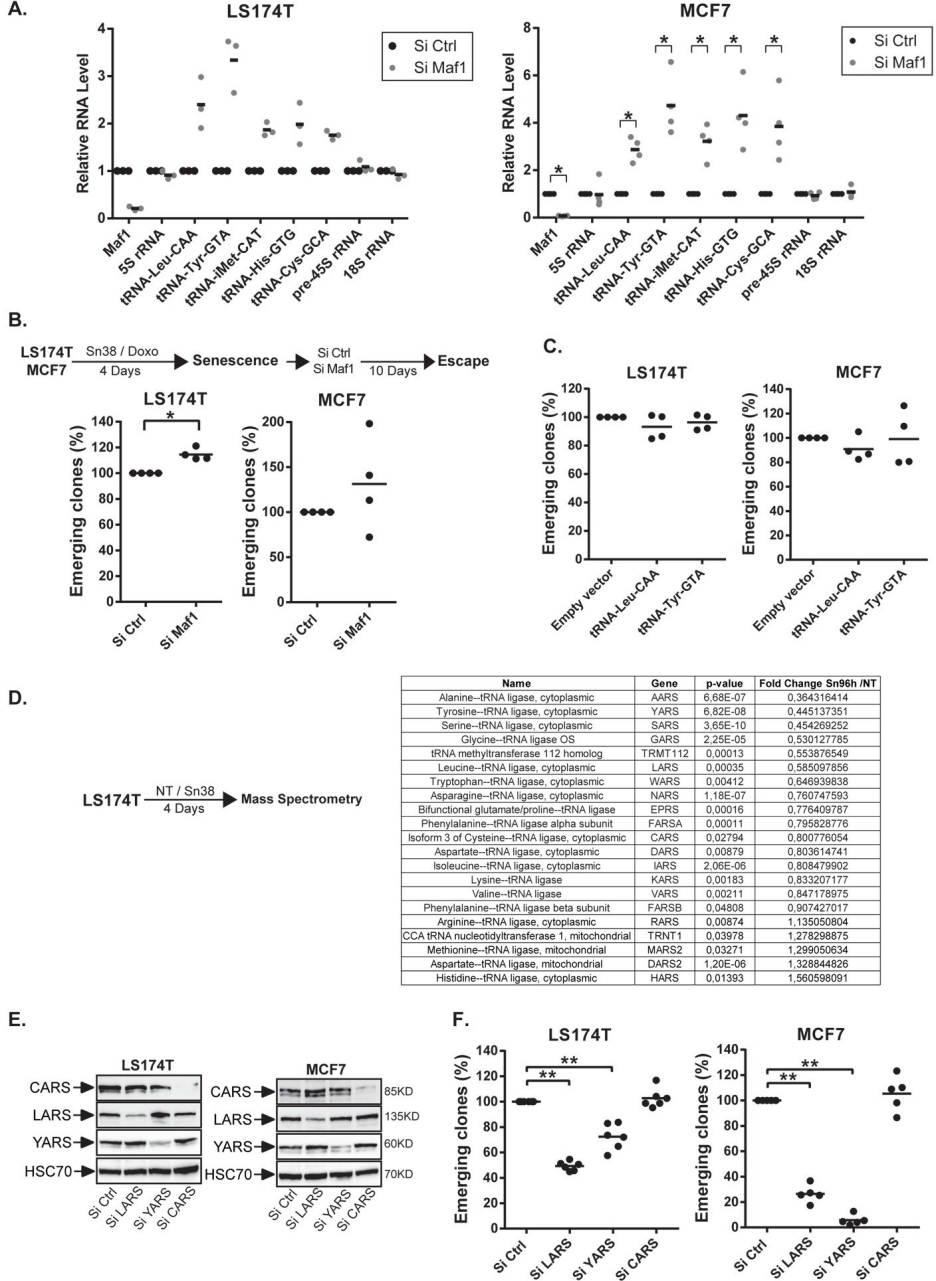
The LARS and YARS aminoacyl-tRNA synthetases are necessary for CIS escape. A. Senescent LS174T and MCF7 cells were transfected with a control siRNA or a siRNA directed against Maf1 during 24 hr and then washed with PBS and stimulated with fresh media. Cell extracts were recovered 2 days after Maf1 depletion and the expression of the indicated RNAs was analyzed by RT-QPCR (LS174T: n = 3; MCF7: n = 4, Kolmogorov-Smirnov test, * = p<0.05). B. Cells were treated and transfected as described above. Nine days after Maf1 inactivation, the number of emerging clones was analyzed (n = 4, Kolmogorov-Smirnov test, * = p<0.05). C. LS174T and MCF7 senescent cells were transduced with an empty vector (pLKO.1) or a vector expressing the tRNA-Tyr-GTA or the tRNA-Leu-CAA. Ten days after, the number of emerging clones was analyzed (n = 4). D. Quantitative proteomic analysis of proteins involved in tRNA biogenesis between growing and LS174T senescent cells (n = 3). E. Senescent LS174T and MCF7 cells were transfected with a control siRNA or a siRNA directed against LARS, YARS or CARS. The depletion of the specifics Aminoacyl-tRNA Synthetases was validated by western blot 2 days after the transfection (n = 2). F. Senescent LS174T and MCF7 cells were transfected with a control siRNA or a siRNA directed against LARS, YARS or CARS for 24 hr. The number of emergent clones was evaluated 9 days later (LS174T n = 6, MCF7 n = 5, Kolmogorov-Smirnov test, ** = p<0.01).

These results suggested to us that CIS escape might be induced either by a global reactivation of RNA pol III activity or by the over-expression of specific tRNAs. One could first speculate that a general activation of tRNA pathways promotes CIS escape by simply increasing protein synthesis. To test this hypothesis, we inactivated MAF1, the RNA Pol III repressor, when senescence was established. This led to a general increase of tRNA expression, both in LS174T and MCF7 cells (Fig 6A). Surprisingly, a weak increase of CIS escape was detected in LS174T cells but no significant effect was observed in the breast cell line (Fig 6B). Inactivating MAF1 with a different siRNA in senescent LS174T or MCF7 cells confirmed that this did not modify senescence escape (S8A Fig). Thus, a general increase of tRNA expression in senescent cells was not sufficient to induce CIS escape. This contrast with the results of Fig 4D which showed that a reduction of RNA Pol III transcription during the early step of senescence is required to stabilize this tumor suppressor mechanism.

According to previous studies showing new functions of the tRNA^Glu^ and tRNA^Arg^ during metastatic spreading [24], specific tRNAs might have also unexpected functions during senescence escape [22,23]. We therefore determined if specific tRNAs could induce CIS escape. To this end, we infected senescent cells with lentivirus expressing the tRNA^Leu^-CAA and the tRNA^Tyr^-GTA. Results presented Fig 6C indicated that this did not modify the number of persistent clones. This could be explained since endogenous tRNAs are probably expressed in large excess. Instead of increasing their expression, we made the hypothesis that tRNA activity might be regulated through the modulation of aminoacyl-tRNA synthetases. These enzymes allow the ligation of amino acid to their compatible tRNAs. We first used mass spectrometry to analyze their expression in our experimental conditions (Fig 6D). We observed that a significant number of tRNA ligases were down-regulated during senescence whereas others were up-regulated, indicating that this suppressive arrest did not induce a general inhibition of these proteins. To indirectly test the role of the tRNA^Leu^-CAA, tRNA^Tyr^-GTA and tRNA^Cys^-GCA, we down-regulated the expression of the leucine (LARS), tyrosine (YARS) and cystein (CARS) ligases (Fig 6E). Results presented Fig 6F indicated that LARS and YARS inactivation reduced senescence escape, both in LS174T and MCF7 cells. No effect was seen when the CARS ligase was inhibited. This effect of YARS and LARS was confirmed with different siRNA sequences (S8B Fig).

Altogether, these results indicate that LARS and YARS are necessary for senescence escape and that this is specific since CARS had no effect on cell emergence.

### LARS and YARS modulate E2F-1 proliferative and apoptotic targets

**Fig 7:**
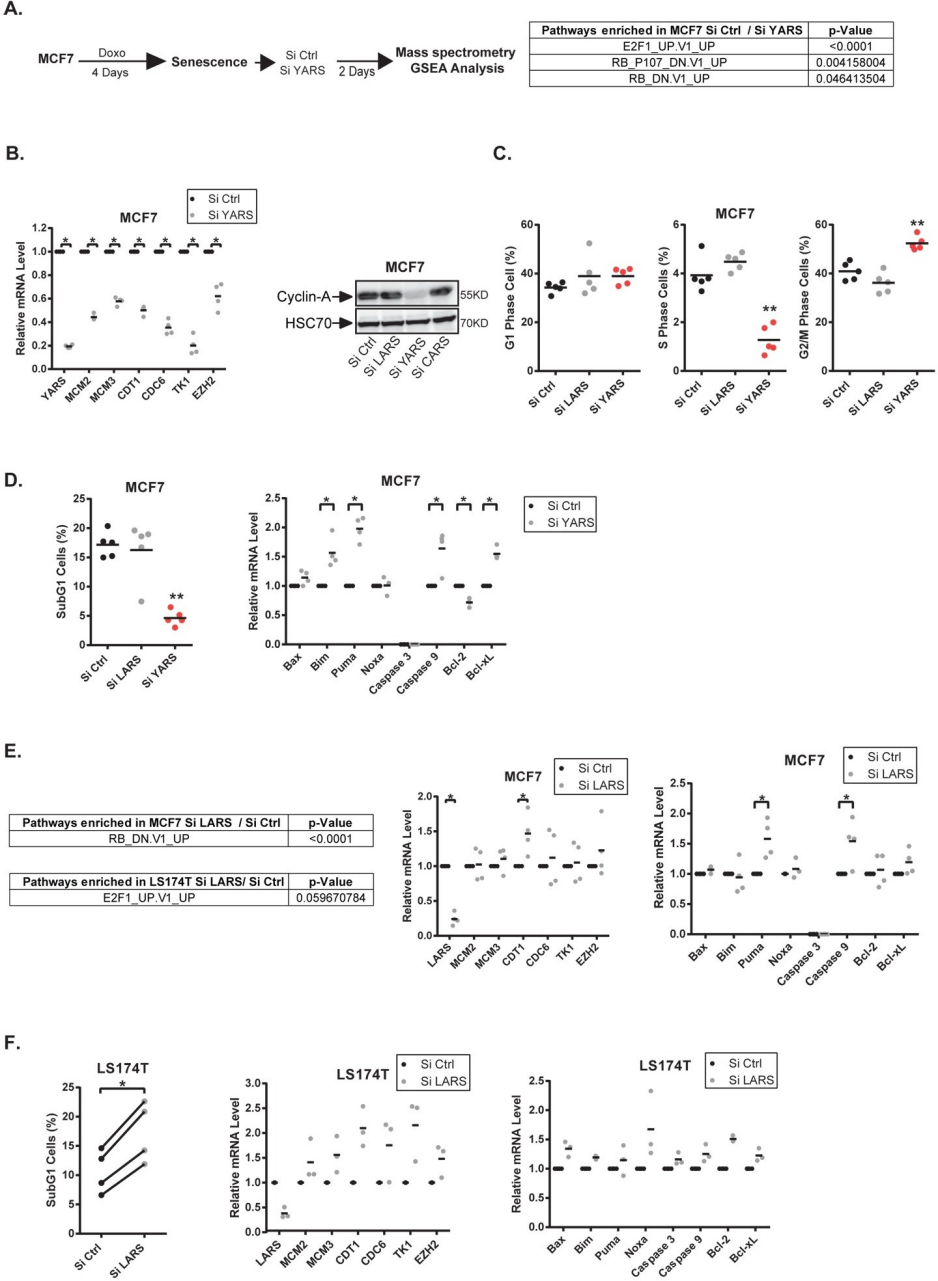
YARS and LARS regulate E2F targets during CIS escape. A. Senescent cells were transfected with a control siRNA or a siRNA directed against YARS. Cell extracts were analyzed by SWATH quantitative proteomics and GSEA analysis 2 days after depletion (n = 3). B. Analysis by RT-QPCR and western blot of E2F1 proliferative targets in MCF7 cells following YARS depletion (n = 4, Kolmogorov-Smirnov test, * = p<0.05, n = 2 for western blot). C. Senescent cells were transfected with a control siRNA or a siRNA directed against YARS or LARS. After two days, FACS analysis was performed to analyze the cell cycle profile of the indicated cells (n = 5, Kolmogorov-Smirnov test, ** = p<0.01). D. Senescent MCF7 cells were transfected as above, FACS analysis was performed to analyze apoptotic cells (n = 5, Kolmogorov-Smirnov test, ** = p<0.01). The expression of the indicated mRNAs was analyzed in parallel by RT-QPCR (n = 4, Kolmogorov-Smirnov test, * = p<0.05). E. MCF7 or LS174T senescent cells were transfected with a control siRNA or a siRNA directed against LARS. Cell extracts were analyzed by SWATH quantitative proteomics and GSEA analysis 2 days after depletion (n = 3). In parallel, the indicated mRNAs were analyzed by RT-QPCR in MCF7 cells following LARS depletion (n = 4, Kolmogorov-Smirnov test, * = p<0.05). F. LS174T senescent cells were transfected as above, FACS analysis was performed to analyze apoptotic cells (n = 4). The expression of the indicated mRNAs was analyzed in parallel by RT-QPCR (n = 3).

To understand how the tRNA ligases could affect cell emergence, we inactivated YARS in MCF7 senescent cells and performed a proteomic analysis after two days of emergence. A significant signature of the Rb and E2F1 pathway was detected (Fig 7A, see all enrichment plot in S9 Fig). This observation was interesting since recent results have connected the deregulation of ribosome biology to the Rb-E2F pathways [34]. To validate this proteomic analysis, we first used MCF7 cells and observed using RT-QPCR experiments that the inactivation of YARS led to a down-regulation of E2F proliferative targets after two days of emergence (Fig 7B, left). This was confirmed with a different siRNA sequence for most tested genes (S8C Fig). Western blot analysis indicated that cyclin A expression was also down-regulated (Fig 7B, right). Interestingly, the down-regulation of this tRNA ligase increased the proportion of cells in the G2/M phase of the cell cycle and reduced S phase entry (Fig 7C).

Besides cell cycle progression, E2F1 is a well-characterized activator of apoptosis. In MCF7 cells, YARS inhibition surprisingly reduced the percentage of apoptotic cells detected during the initial steps of senescence escape (Fig 7D left). RT-QPCR experiments showed that pro-apoptotic members of the Bcl-2 family such as Puma or Bim were up-regulated as well as the Caspase 9 mRNA (Fig 7D right). All these genes have been previously described as E2F1 targets. However, these results also showed that Bcl-xL was up-regulated, suggesting that this survival protein inhibited its pro-apoptotic counterparts. Therefore, in MCF7, YARS inactivation prevented CIS escape and this was associated with a down-regulation of E2F1 proliferative targets. Note however that this was not observed in LS174T cells where these cell cycle and apoptotic targets were not significantly modified (S10A–S10C Fig).

Following LARS inhibition, the GSEA analysis also detected a deregulation of the Rb-E2F1 pathway (Fig 7E left, see all enrichment plot in S9 Fig). E2F1 proliferative targets were not increased in MCF7 cells but an increased expression of Puma and Caspase 9 was detected when LARS was inhibited. In LS174T cells, an increased proportion of dying cells was observed and apoptotic genes such as Noxa were up-regulated (Fig 7F right). In addition, an increased expression of E2F proliferative target genes was observed and this effect was confirmed with a different siRNA sequence targeting LARS (Figs 7F—middle panel—and S8D).

Altogether, these results indicate that LARS and YARS regulate the E2F1 pathway during senescence escape. However, this effect is heterogeneous and depends on the cell line, since apoptotic and proliferative genes were regulated differently by the two ligases and different observations were made in MCF7 and LS174T cells.

## Discussion

Several studies have reported that senescence is much more dynamic than initially expected. How this heterogeneity is regulated remains largely unknown. It can be related to specific transcriptional activities, to the maintenance of epigenetic marks, to the variability of the SASP or to a specific oncogenic background in the case of cancer cells. Single-cell variability is expected to generate subpopulations of cells that enter distinct states of light or deep senescence and as a result some cells will escape chemotherapy more easily. In this study, we extend these observations and propose that the strength of CIS is related to the expression of specific tRNAs and aminoacyl-tRNA synthetases.

It is known that perturbations of ribosome biogenesis induce suppressive pathways. Ribosomal proteins such as L11 can interact with hdm2 to activate p53 and induce cell cycle arrest [35]. These proteins also controlled senescence in a p53-independent manner since the RPS14 protein can interact with cdk4 to prevent its activity. This inhibits Rb phosphorylation and induces senescence as a response to an abnormal ribosome biogenesis [34]. Our results indicate that tRNA transcription is inhibited during the initial step of the suppressive arrest but that emergent cells reactivate this activity to allow senescence escape. This effect is specific and not related to a general activation of RNA Pol III since only specific tRNAs are up-regulated during emergence. Of the aminoacyl-tRNA synthetases tested, only the LARS and YARS tRNA ligases allowed senescence escape. Importantly, the CARS ligase did not affect emergence and the expression of the corresponding tRNA^Cys^ was not modified. Given the complexity of tRNA biology, it remains to be determined on a larger scale if other tRNAs and ARSs are involved in cell emergence. We speculate that this will be the case and that different pools of tRNAs and ligases will regulate senescence pathways. Interestingly, recent results have shown that senescence prevents the ribosomal readthrough of stop codons and that cells that escape this suppressive mechanism have an abnormal translation termination [36]. Accordingly, we speculate that a specific expression of tRNAs and ligases might regulate the expression of suppressive or oncogenic proteins. This could explain senescence heterogeneity and the ability of cells to remain definitely arrested or conversely to restart proliferation.

Further experiments are necessary to determine how tRNAs and ARSs regulate CIS and cell emergence. As described above [34], we have observed that these proteins deregulate the E2F1 pathway. Further experiments are now necessary to understand how YARS and LARS regulate E2F1 and if this is also mediated by RPS14 and cdk4 inhibition, but our results confirm that this signaling pathway is a main target of ribosome biology. It should also be noted that some aminoacyl-tRNA synthetases have adopted new functions during evolution, beyond protein synthesis. For instance, the leucyl-tRNA synthetase can induce mTOR activity upon leucine stimulation [37]. The Trp-tRNA synthetase can interact with DNA-PK and PARP to activate p53 [38]. It has also been recently reported that the seryl tRNA synthetase interacts with the POT1 member of the telomeric shelterin complex and that this accelerates replicative senescence [39]. Thus, we can speculate that the LARS and YARS tRNA ligases have acquired new functions that somehow allow senescence escape.

New results have also described unexpected functions for tRNAs [22,23]. Recent findings show that tRNA^Glu^ and tRNA^Arg^ are over-expressed in metastatic cells and that their inactivation reduces *in vivo* metastasis [24]. These tRNAs induce a profound change in the proteome, with enrichment of proteins containing the GAA and GAG codons in the corresponding mRNA. This effect is specific, as it is not seen with other tRNAs. Santos et al. have recently reported that mutant tRNAs that mis-incorporate Serine instead of Alanine induce cell transformation [40]. It has been proposed that a change in the pool of available tRNAs can lead to statistical errors in tRNA load within the ribosomal subunits [22,23]. This generates a random proteome known as a statistical proteome, which represents mutated proteins or proteins with a novel sequence not completely coded by the genome [41,42]. It will be interesting to determine if the variability of the SASP might be explained by the utilization of different tRNA pools. Although this remains to be demonstrated, it leads to the hypothesis that subpopulations of senescent cells might express specific tRNAs (or tRNA ligases) and that this will modify the composition and activity of secretomes presenting distinct, specific inflammatory activities.

Further experiments are therefore needed to determine if different pools of tRNAs and aminoacyl-tRNA synthetases are expressed in response to oncogenic insults, chemotherapy or during aging. We speculate that each type of suppressive arrest might lead to the expression of specific tRNAs and ARSs and that this might explain the specificity of these responses. In addition to epigenetic and transcriptional regulations, we therefore propose that the heterogeneity of tRNAs and ligases expression also leads to distinct states of light or deep senescence. This observation provides a rationale to further study the different facets of senescence responses and their links with chemotherapy resistance.

## Materials and methods

See also the supplementary files S1 Methods and S1 Table.

### Ethics statement

As required by the French Committee for the Protection of Human Subjects, informed written consent was obtained from patients and the board of the Nantes hospital ethic committee approved protocols under the Therex Program (2012-A00682-41).

### Cell lines, senescence induction, senescence escape and terminology

LS174T and MCF7 cell lines were obtained from the American Type Culture Collection. Cell lines were authenticated by STR profiling and were regularly tested to exclude mycoplasma contamination. To induce senescence, cell lines were treated for 96 hr in RPMI medium containing 3% FBS with Sn38 (5 ng/ml, LS174T) or Doxorubicin (25 ng/ml, MCF7). Control cells were treated with the corresponding DMSO vehicle for 96hr as a control. To promote senescence escape cells were washed with PBS and stimulated with fresh medium containing 10% FBS for 7 (RT-qPCR analysis, Western Blot, SA-β galactosidase staining) or 10 days (evaluation of emerging clone number). In this manuscript, senescence escape is also described as emergence or re-proliferation of escaping clones; cells that restart proliferation are called indifferently escaping clones, emergent cells or persistent cells: all correspond to cells that have escaped senescence.

### Treatments

Cells were treated with the following drugs:

Torin-1 (Cell Signaling, 14379): 15 nM, Rapamycin (Santa-Cruz, sc-3504): 5 nM, Tunicamycin (Santa-Cruz, sc-3506): 0.1 to 8 μg/ml, Thapsigargin (Santa-Cruz, sc-24017): 10 to 50 nM. ABT-263 or Navitoclax (Clini Sciences, A3007): 5μM.

### SiRNA transfection

Cells were transfected with 50 nM of a smart pool of 4 small interfering RNAs against p21(CDKN1A) (ON-TARGET plus Human CDKN1A (1026) Dharmacon, L-00341-00-0005), Raptor (ON-TARGETplus Human RPTOR (57521), Dharmacon, L-004107-00-0005), Maf1 (ON-TARGET plus Human MAF1 (84232), Dharmacon, L-018603-01-0005), BRF1 (ON-TARGET plus human BRF1 (2972), Dharmacon, L-017422-00-0005), LARS (ON-TARGETplus Human LARS (51520), L-010171-00-0005), YARS (ON-TARGETplus Human YARS (8565), L-011498-00-0005), CARS (ON-TARGETplus Human CARS (833), L-010335-01-0005) and prevalidated control siRNA (Dharmacon, D-001810-10-20) using DharmaFect-4 (Dharmacon, T-2004-03). Note that the siRNA concentration was reduced to 12,5nM for the tRNA ligases to reduce cell toxicity. Main results were also validated with a different individual small interfering RNA against TSC2 (ON-TARETplus Human TSC2 (7249), Dharmacon, J-003029-10-0020), LARS (ON-TARETplus Human LARS (51520), Dharmacon, J-010171-05-0020), YARS (ON-TARETplus Human YARS (8565), Dharmacon, J-011498-05-0020), BRF1(ON-TARETplus Human BRF1 (2972), Dharmacon, J-017422-05-0020), MAF1 (ON-TARETplus Human MAF1 (84232), Dharmacon, J-018603-11-0020).

### ShRNA, lentiviruses and cell transduction

pLKO.1-TSC2 was a gift from Do-Hyung Kim (Addgene plasmid #15478), pLKO-TRC2 (scramble ShRNA, Sigma mission, SH216). For the generation of lentiviruses, 293 cells were cotransfected with the packaging plasmids (pMDLg/pRRE, pRSV-Rev, and PMD2.G) and the different PLKO plasmids by lipofectamine 2000 for 24 hr. After 24 hr, the medium was replaced with fresh medium. After 48 hr, virus-containing supernatant was collected and centrifuged for 5 minutes at 300g and filtered through 0.45 μm. For the transduction, 2.5 ml were used to transduce LS174T and MCF7 senescent cells in the presence of 4 μg/ml Polybrene (Santa Cruz). After 24h, the media was replaced. For tRNA over-expression, the TRY-GTA5-3 tRNA-Tyr and TRL-CAA2-1 tRNA-Leu sequence were cloned in the pLKO.1 vector.

### Cell cycle analysis

250 000 cells were incubated with 150 μL of solution A (trypsin 30 μg/mL, Sigma) for 10 min at room temperature in the dark. Then, 125μL of solution B (trypsin inhibitor 0,5 mg/mL, RNAse A 0,1 mg/mL, Sigma) was added for 10 min in the dark. Finally, cells were incubated with 125 μL of solution C (propidium iodide 0,6 mM, spermine tetrahydrochloride 3,3 mM, Sigma) for 10 min at 4°C. All the solutions were prepared in a storage buffer pH 7,6 containing 3,4 mM sodium citrate 2H_2_0 (Sigma), 0,1% Igepal CA-630 (Sigma), 3 mM spermine tetrahydrochloride and 1mM de tris-aminomethane. Cells were then analyzed using a BD LSRII, 30 000 events were recorded per sample.

### Patient-derived organoids

Breast tumors from patients who underwent surgical tumor resection at the ICO Paul Papin Cancer Center were processed through a combination of mechanical disruption and enzymatic digestion by collagenase to generate patient-derived organoids (PDO) as described (33). Briefly, isolated cells were plated in adherent basement membrane extract drops (BME type 2, R&D systems, 3533-010-02) and overlaid with optimized breast cancer organoid culture medium. Medium was changed every 4 days and organoids were passaged every 1–3 weeks. Organoids were treated by 2 cycles of doxorubicin (25 ng/ml for organoid #1, 50 ng/ml for organoid #2) for 96 hrs. At the end of the first cycle, the medium was changed and organoids were incubated 3 days without chemotherapy before the second cycle. Following the 2 cycles, the medium was replaced and changed every 3–4 days to analyze chemotherapy escape. As required by the French Committee for the Protection of Human Subjects, informed consent was obtained from patients and the local ethic committee approved protocols under the Therex Program (2012-A00682-41).

## Supporting information

S1 Fig Senescence induction following chemotherapy treatment in LS174T and MCF7 cells. (PDF)

S2 Fig Activation and role of the mTOR pathway during senescence escape. (PDF)

S3 Fig mTOR inhibition increases senescence stability. (PDF)

S4 Fig Protein stress limits CIS escape. (PDF)

S5 Fig BRF1 down-regulation maintains senescence. (PDF)

S6 Fig A transient cell cycle arrest induced by chemotherapy treatment is not associated with a profound change in tRNA expression. (PDF)

S7 Fig The senolytic agent ABT-263 blocks chemotherapy escape of breast cancer organoids and cancer cell lines. (PDF)

S8 Fig Confirmation of the effects of Maf1, LARS and YARS inhibition with different siRNAs. (PDF)

S9 Fig Enrichment plot of the proteomic signatures. (PDF)

S10 Fig Effect of YARS inhibition in LS174T colorectal cells. (PDF)

S1 Methods Supplementary Methods. (DOCX)

S1 Table Supplementary Primers List. (XLSX)
