# Supplementary material for: tRNA biogenesis and specific aminoacyl-tRNA synthetases regulate senescence stability under the control of mTOR
Source: PLoS Genet. 2021 Dec 20;17(12):e1009953. doi: 10.1371/journal.pgen.1009953 (PMC8722728; doi:10.1371/journal.pgen.1009953)
Supplement: S1 Fig — (PDF) [file pgen.1009953.s001.pdf]

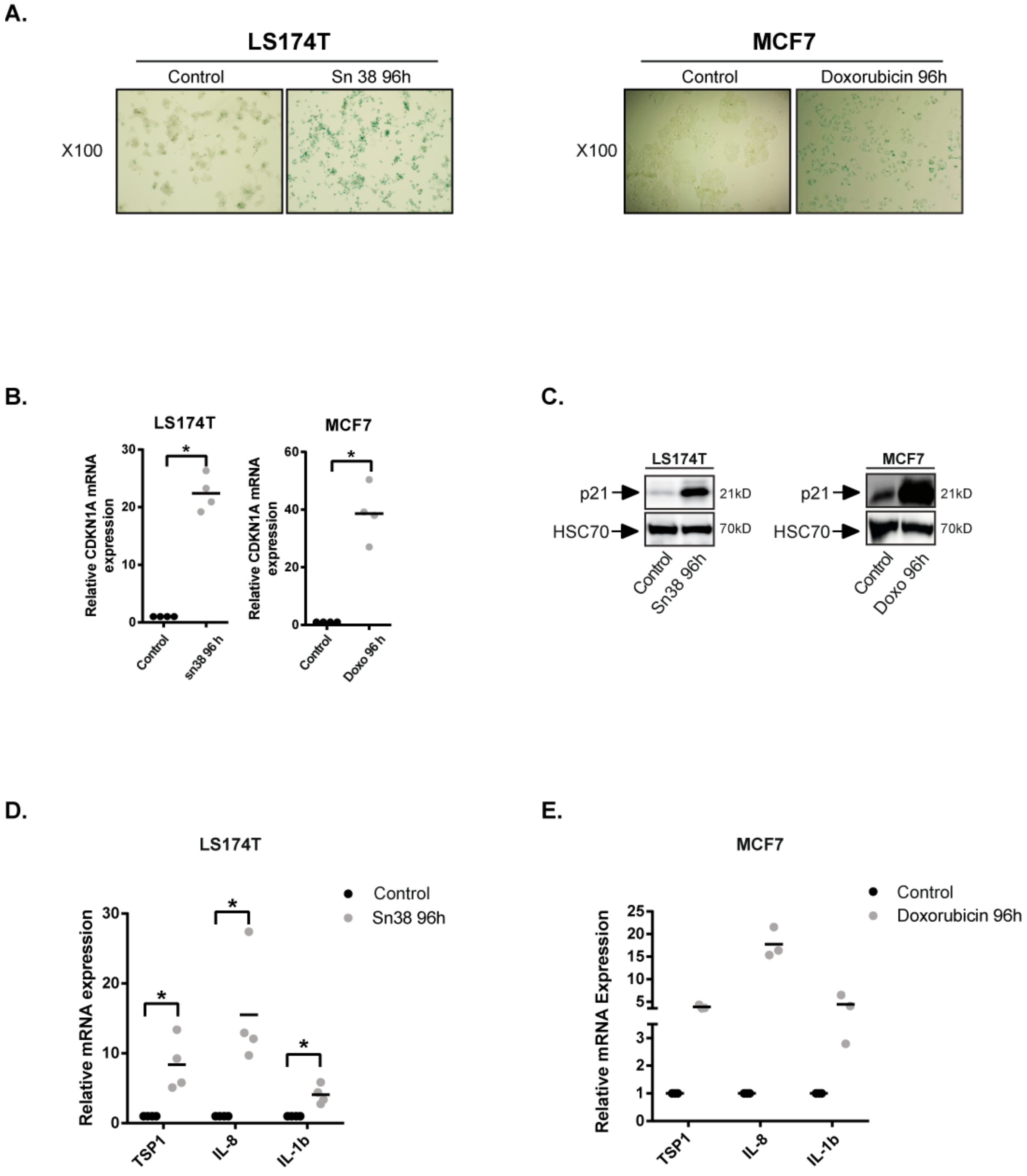

S1 Fig: Senescence induction following chemotherapy treatment in LS174T and MCF7 cells.
A. Representative images of SA-β galactosidase staining 96 hours after the treatment or control (cells treated with the DMSO vehicle) in LS174T colorectal and MCF7 mammary cells. (LS174T n= 3, MCF7 n=2, sn38 is a topoisomerase I inhibitor) **B**. Analysis by RT-QPCR of CDKN1A mRNA expression in MCF7 and LS174T cells after 96 hours of treatment. (n=4, Kolmogorov-Smirnov test, * = $p<0.05$) **C**. Analysis by western blot of p21 expression in MCF7 and LS174T cells after 96 hours of treatment. (LS174T n= 3, MCF7 n=1) **D**. Analysis by RT-QPCR of indicated mRNA expression in LS174T cells after 96 hours of treatment with Sn38 (n=4, Kolmogorov-Smirnov test, * = $p<0.05$). **E**. Analysis by RT-QPCR of indicated mRNA expression in MCF7 cells after 96 hours of treatment with Doxorubicin (n=3).
