# Supplementary material for: tRNA biogenesis and specific aminoacyl-tRNA synthetases regulate senescence stability under the control of mTOR
Source: PLoS Genet. 2021 Dec 20;17(12):e1009953. doi: 10.1371/journal.pgen.1009953 (PMC8722728; doi:10.1371/journal.pgen.1009953)
Supplement: S2 Fig — (PDF) [file pgen.1009953.s002.pdf]

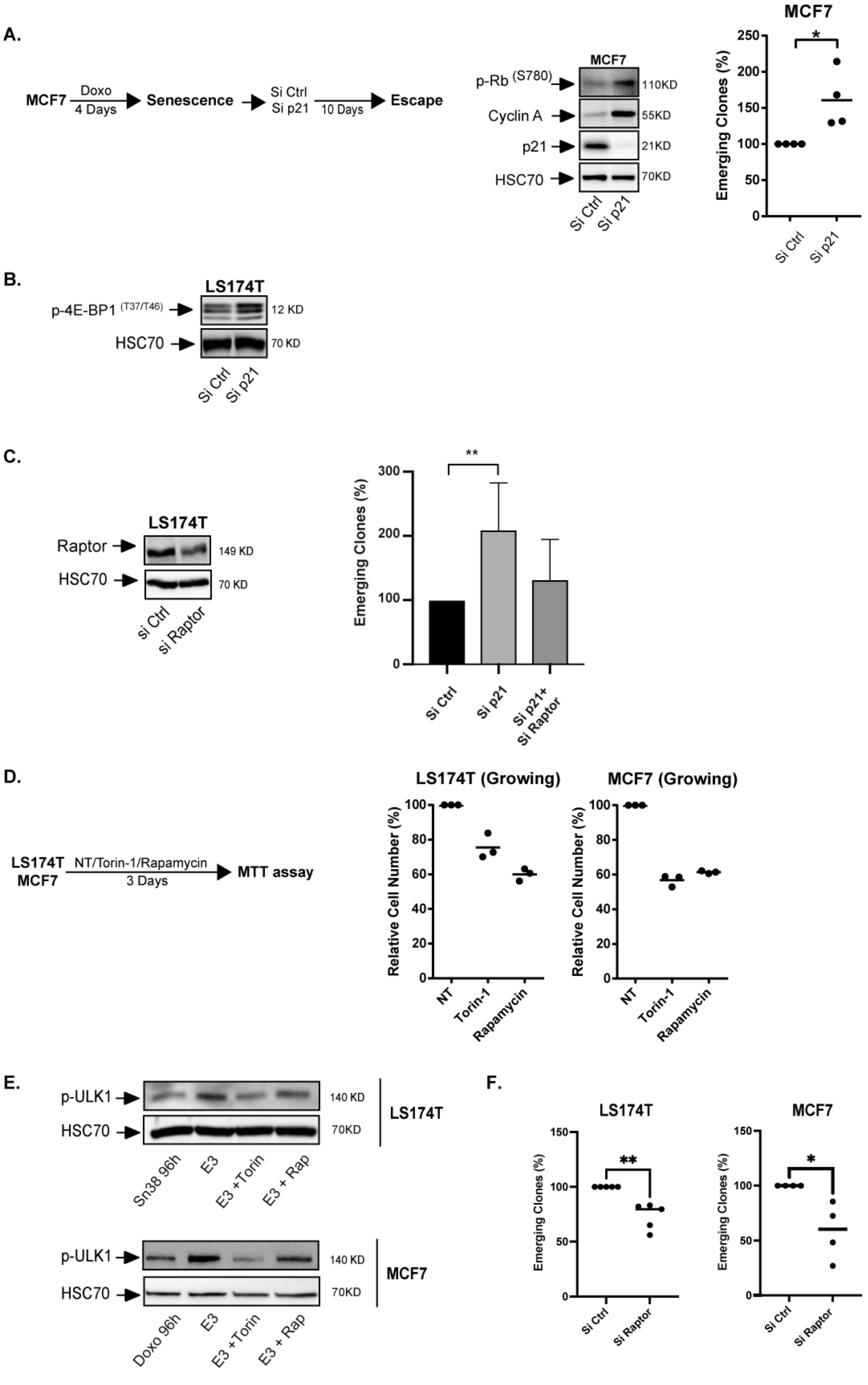

S2 Fig: Activation and role of the mTOR pathway during senescence escape.

A. Senescent MCF7 cells were transfected with a control siRNA or a siRNA directed against p21 for 24 h and persistent cells were then generated by adding 10% FBS. p21 down-regulation as well as the expression of cyclin A and Rb phosphorylation were evaluated by Western blot two days after the depletion. Emergence was evaluated after 10 days (n=4, Kolmogorov-Smirnov test, * = p<0.05, n=3 for western blot). **B**. LS174T were treated as above and cell extracts were recovered 2 days after p21 depletion. The expression of the indicated proteins was analyzed by western blot (n= 2) **C.** Number of emerging LS174T clones analyzed after p21 or p21 and raptor inactivation (n=5, Kolmogorov-Smirnov test ** = p<0.01). Raptor inactivation was validated by western blot 2 days after the transfection (n=3). **D.** LS174T and MCF7 cells were treated or not with mTOR inhibitors (Torin-1:15nM ; Rapamycin:5nM), and cell viability was analyzed by MTT assay after 3 days (n=3) **E**. LS174T and MCF7 senescent cells were generated as above and emergence was induced by adding 10% FBS in the presence or absence of mTOR inhibitors (Rapamycin: 5nM, Torin-1: 15nM). Cell extracts were recovered after 3 days and the expression of the indicated proteins was analyzed by western blot. (n=2). F. Number of emerging clones analyzed after Raptor inactivation by siRNA transfection in LS174T and MCF7 senescent cells. (MCF7 n=4, LS174T n=5, Kolmogorov-Smirnov test * = p<0.05, **= p<0.01).
