# Supplementary material for: tRNA biogenesis and specific aminoacyl-tRNA synthetases regulate senescence stability under the control of mTOR
Source: PLoS Genet. 2021 Dec 20;17(12):e1009953. doi: 10.1371/journal.pgen.1009953 (PMC8722728; doi:10.1371/journal.pgen.1009953)
Supplement: S3 Fig — (PDF) [file pgen.1009953.s003.pdf]

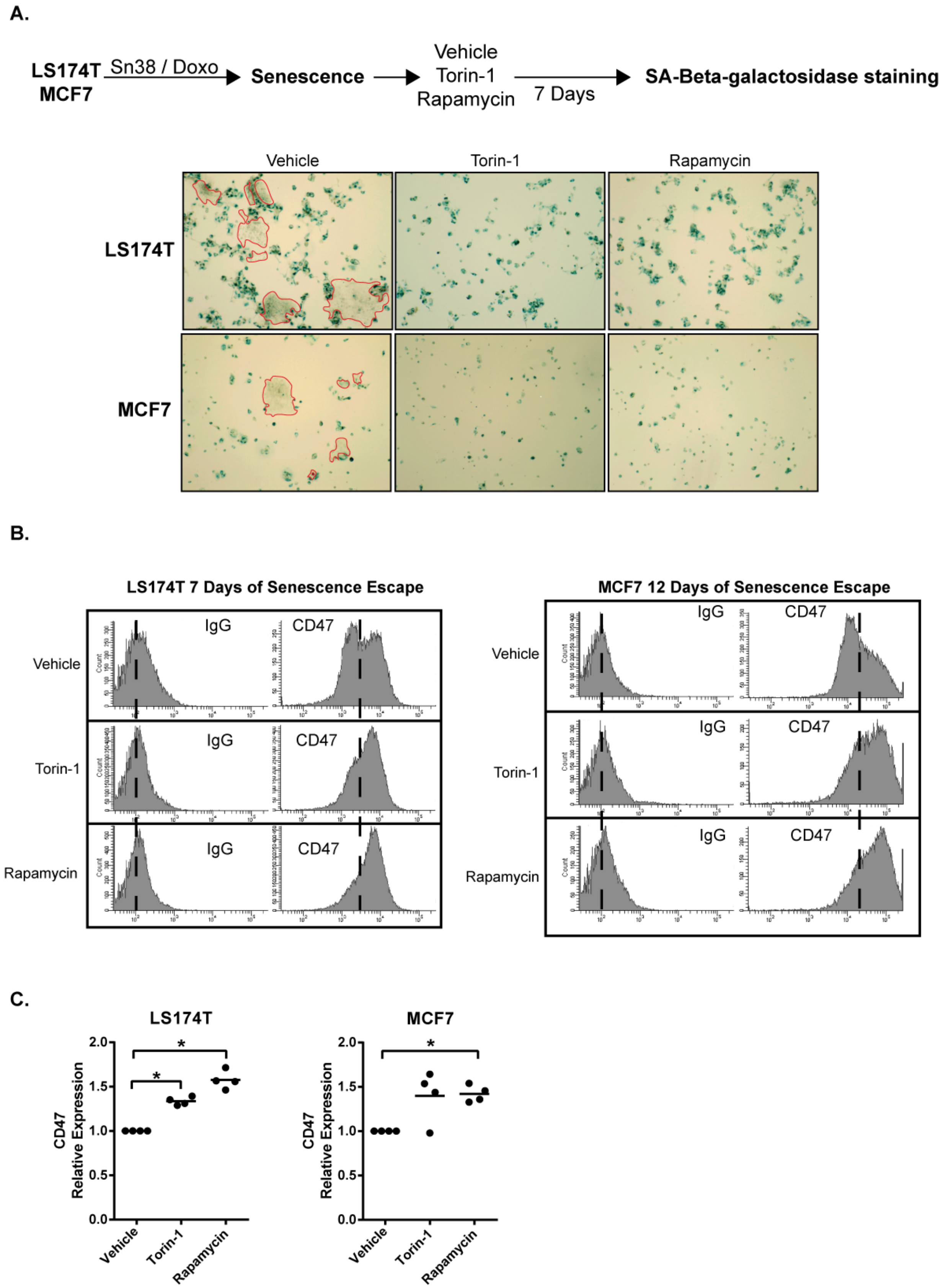

S3 Fig: mTOR inhibition increases senescence stability.

A. Representative images of SA-β galactosidase staining 7 days after mTOR inhibition in LS174T (n=3) and MCF7 (n=2) senescent cells. Growing persistent cells are underlined in red.

B. and C. LS174T and MCF7 senescent cells were treated with mTOR inhibitors (Torin-1 :15nM, Rapamycin 5nM) or with the vehicle (DMSO) and CD47 extracellular expression was analyzed by flow cytometry on emerging cells, after 7 to 12 days. **B:** Representative histograms of extracellular staining of CD47. **C:** Quantification of CD47 relative expression of cells treated as above (n=4, Kolmogorov-Smirnov test, * = $p<0.05$).
