# Supplementary material for: tRNA biogenesis and specific aminoacyl-tRNA synthetases regulate senescence stability under the control of mTOR
Source: PLoS Genet. 2021 Dec 20;17(12):e1009953. doi: 10.1371/journal.pgen.1009953 (PMC8722728; doi:10.1371/journal.pgen.1009953)
Supplement: S4 Fig — (PDF) [file pgen.1009953.s004.pdf]

A.

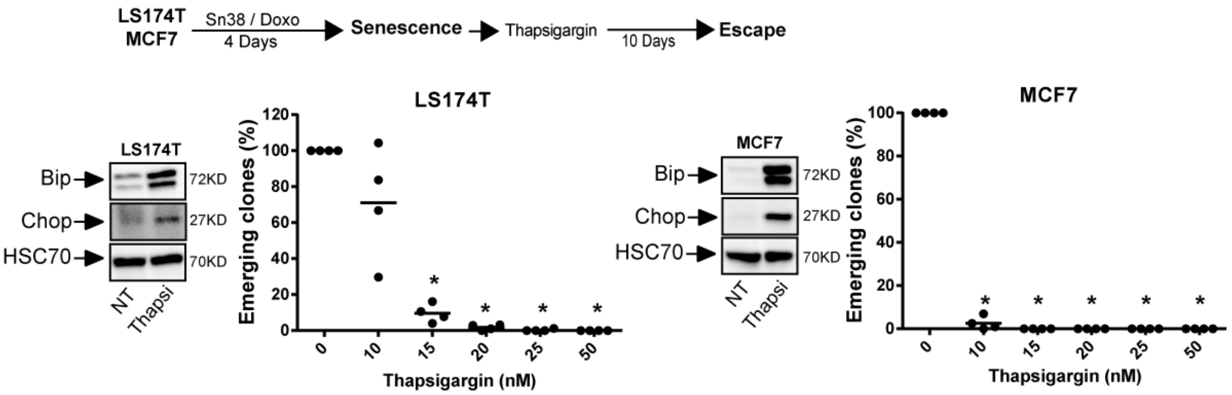

B.

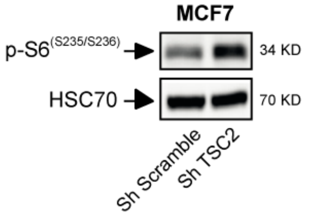

C.

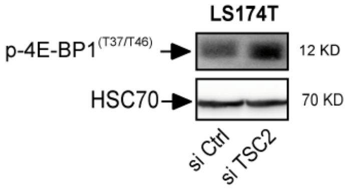

S4 Fig: Protein stress limits CIS escape

A. Senescent cells were treated with increasing concentrations of thapsigargin and the number of emerging clones was evaluated 10 days later (n=4, Kolmogorov-Smirnov test,* = $p<0.05$). The induction of UPR was validated by Western blot after 24h using a 10 nM concentration (n=2) **B**. Following senescence induction, MCF7 cells were transduced with a control shRNA or a shRNA directed against TSC2. mTORC1 activity was evaluated through S6 phosphorylation by western blot 2 days after transduction (n= 1). **C**. Following senescence induction, LS174T cells were transfected with a control siRNA or a siRNA directed against TSC2. mTORC1 activity was evaluated through 4E-BP1 phosphorylation by western blot two days after transfection (n=2).
