# Supplementary material for: tRNA biogenesis and specific aminoacyl-tRNA synthetases regulate senescence stability under the control of mTOR
Source: PLoS Genet. 2021 Dec 20;17(12):e1009953. doi: 10.1371/journal.pgen.1009953 (PMC8722728; doi:10.1371/journal.pgen.1009953)
Supplement: S5 Fig — (PDF) [file pgen.1009953.s005.pdf]

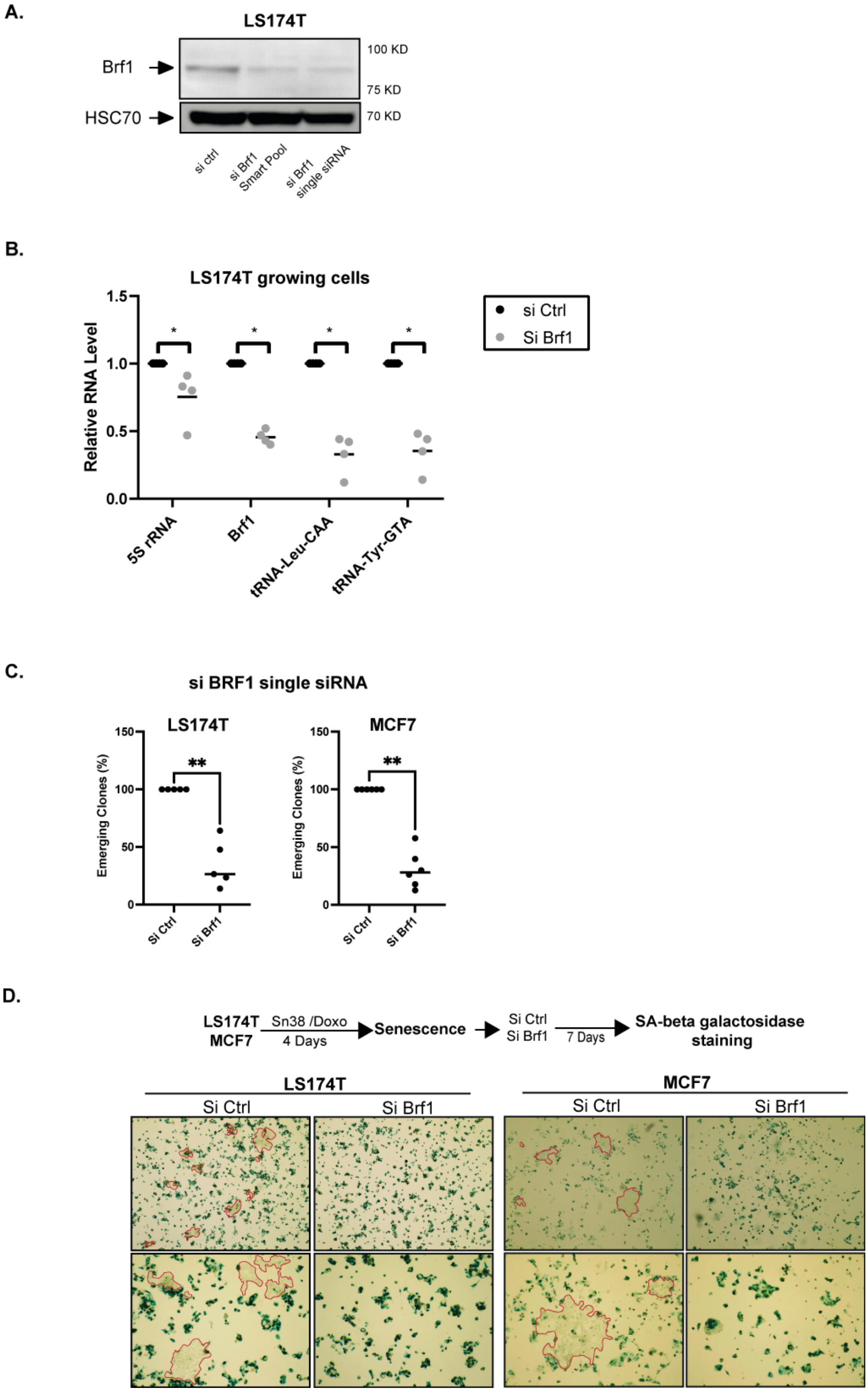

S5 Fig: BRF1 down-regulation maintains senescence

A. LS174T growing cells were transfected with a control siRNA or a siRNA directed against Brf1, either with a smartpool of siRNA or a single siRNA. Brf1 inactivation was validated by western blot 72h after the transfection. **B**. LS174T growing cells were transfected with a control siRNA or a single siRNA directed against Brf1. Cell extracts were recovered 30 hours after Brf1 depletion and the expression of the indicated RNAs was analyzed by RT-QPCR (LS174T n=4, Kolmogorov-Smirnov test * = $p<0.05$). **C**. Senescence was induced by treating respectively LS174T and MCF7 cells with sn38 or doxorubicin during 96 hours. Cells were then washed with PBS and transfected with a control siRNA or a single siRNA directed against Brf1 for 24 hr. The number of emerging clones was analyzed ten days following Brf1 depletion (LS174T n=5, MCF7 n=6 Kolmogorov-Smirnov test ** = $p<0.01$). **D**. Representative images of SA-β galactosidase staining 7 days after BRF1 inactivation in LS174T and MCF7 senescent cells. Growing persistent cells are underlined in red (n=3).
