# Supplementary material for: tRNA biogenesis and specific aminoacyl-tRNA synthetases regulate senescence stability under the control of mTOR
Source: PLoS Genet. 2021 Dec 20;17(12):e1009953. doi: 10.1371/journal.pgen.1009953 (PMC8722728; doi:10.1371/journal.pgen.1009953)
Supplement: S6 Fig — (PDF) [file pgen.1009953.s006.pdf]

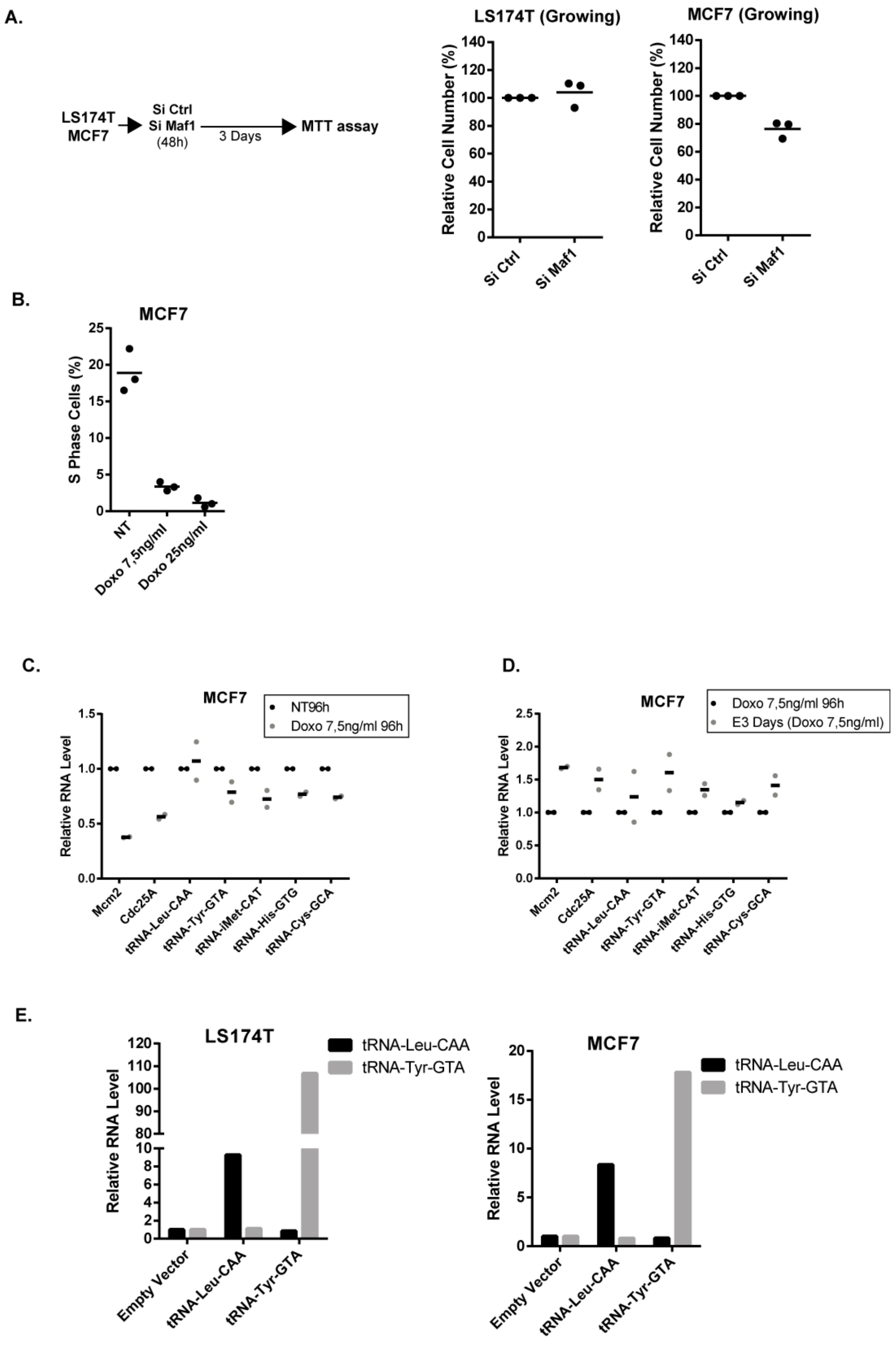

S6 Fig: A transient cell cycle arrest induced by chemotherapy treatment is not associated with a profound change in tRNA expression.

A LS174T and MCF7 cells were transfected with a control siRNA or a si RNA directed against Maf1 for 48h. Cell viability was analyzed by MTT assay after 3 days (n=3). **B.** MCF7 cells were treated or not with 7,5ng/ml or 25ng/ml of Doxorubicin during 4 days. FACS analysis was then performed to analyze the percentage of cells in S phase (n=3). **C.** RT-QPCR anlaysis of the indicted proliferative markers (Mcm2 and Cdc25a) and tRNAs in MCF7 cells treated or not with Doxorubicin (7,5ng/ml) during 4 days (n=2). (**D**) RT-QPCR analysis of the expression of the indicated RNAs in MCF7 cells. Cells have been treated for 4 days with Doxorubicin (7,5ng/ml) and emergence was induced during 3 days by 10% FBS (E3, n=2). **E**. LS174T and MCF7 senescent cells were transduced with an empty vector (pLKO.1) or a vector expressing tRNA-Tyr-GTA or tRNA-Leu-CAA. 10% FBS was then added for two days and RNA expression was analyzed by RT-QPCR.
