# Supplementary material for: tRNA biogenesis and specific aminoacyl-tRNA synthetases regulate senescence stability under the control of mTOR
Source: PLoS Genet. 2021 Dec 20;17(12):e1009953. doi: 10.1371/journal.pgen.1009953 (PMC8722728; doi:10.1371/journal.pgen.1009953)
Supplement: S7 Fig — (PDF) [file pgen.1009953.s007.pdf]

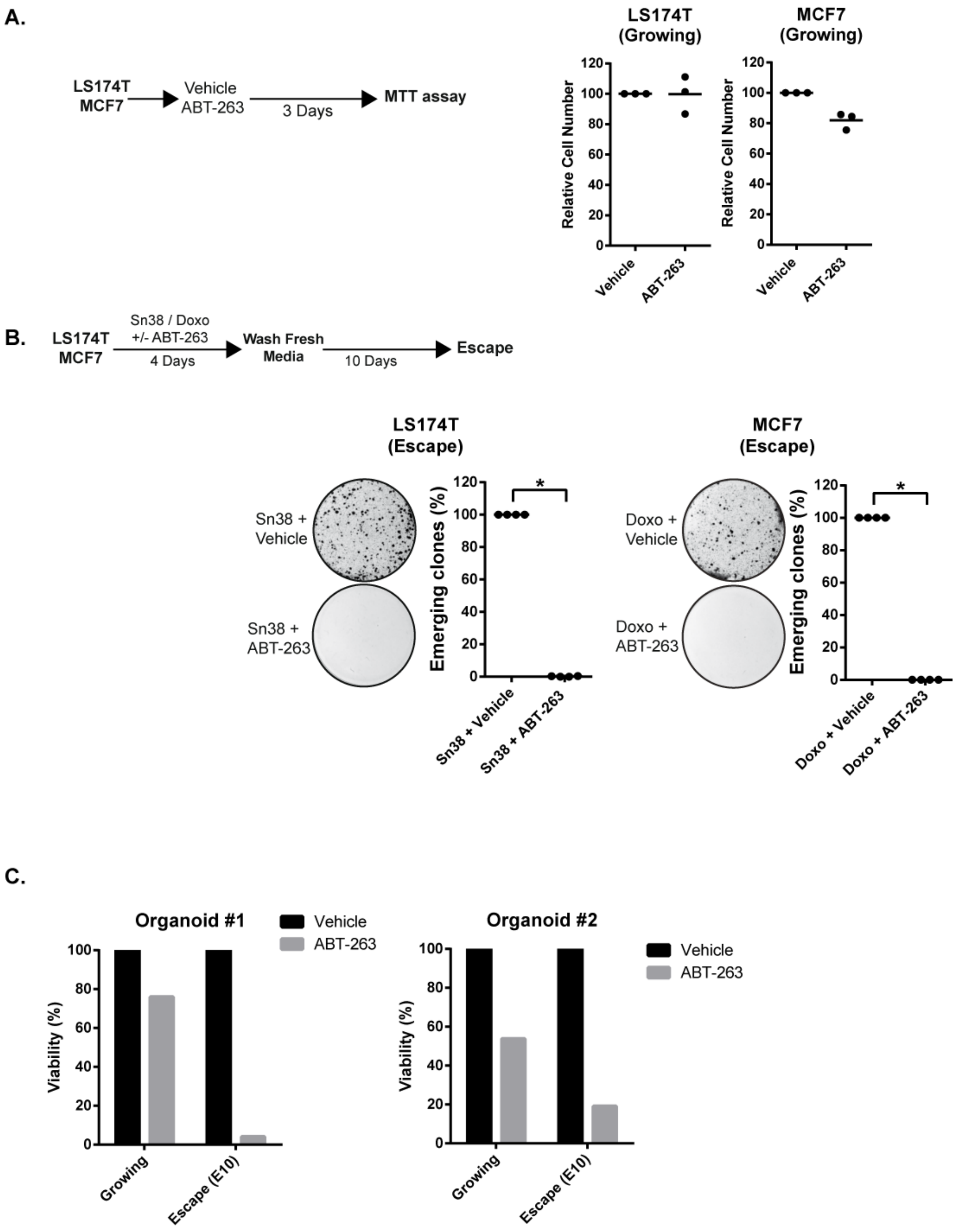

S7 Fig: The senolytic agent ABT-263 blocks chemotherapy escape of breast cancer organoids and cancer cell lines.
A. Evaluation of the viability of MCF7 growing cells treated or not for 72h with ABT-263 (5μM) by MTT assay (n=3). **B.** LS174T and MCF7 cells were treated respectively with sn38 or doxorubicin and Vehicle (DMSO) or ABT-263 (5μM) during 96h. Then, cells were washed with PBS and stimulated with fresh medium containing 10% FBS. The number of emerging clones was evaluated 10 days later after Crystal violet staining (n=4, Kolmogorov-Smirnov test, * = $p<0.05$). **C.** Analysis of the viability of PDOs treated with ABT-263 (5μM), either in growing conditions or when added in combination with doxorubicin. Proliferation was evaluated 10 days after the treatment.
