# Supplementary material for: tRNA biogenesis and specific aminoacyl-tRNA synthetases regulate senescence stability under the control of mTOR
Source: PLoS Genet. 2021 Dec 20;17(12):e1009953. doi: 10.1371/journal.pgen.1009953 (PMC8722728; doi:10.1371/journal.pgen.1009953)
Supplement: S8 Fig — (PDF) [file pgen.1009953.s008.pdf]

*Single siRNA confirmation*

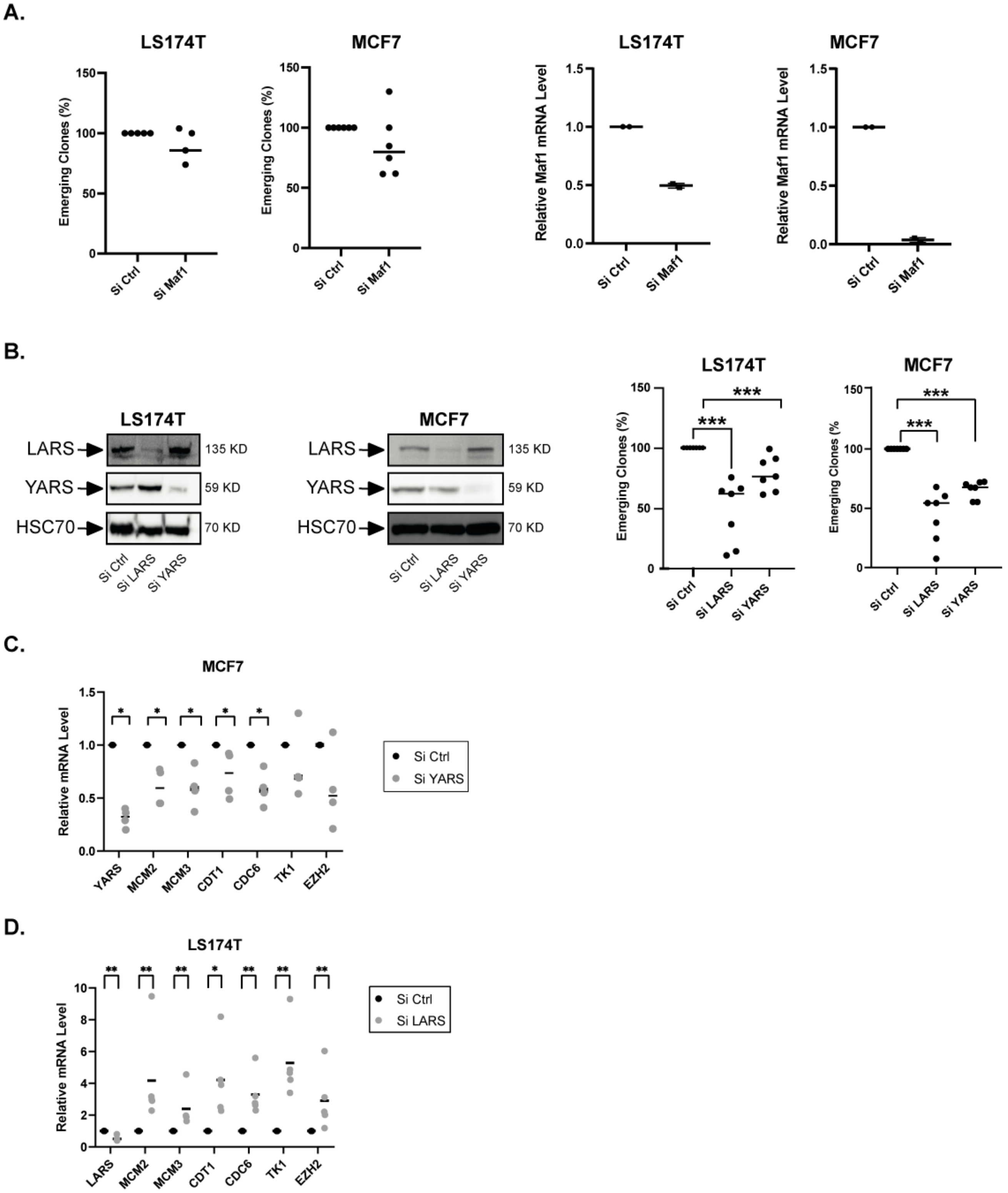


**S8 Fig: Confirmation of the effects of Maf1, LARS and YARS inhibition with different siRNAs.**

**A**. Senescence was induced by treating LS174T and MCF7 cells respectively with sn38 or doxorubicin during 96 hours. Cells were then washed with PBS and transfected with a control siRNA or a single siRNA directed against MAF1 for 24 hr and senescence escape was generated by adding 10% FBS. The number of emerging clones was analyzed following MAF1 inactivation (after 7 days for LS174T cells n=4, after 9 to 10 days for MCF7 n=6). The inhibition of Maf1 expression was verified by RT-QPCR. **B**. Senescent LS174T and MCF7 cells were transfected with a control siRNA or a single siRNA directed against LARS or YARS. The depletion of the specifics Aminoacyl-tRNA Synthetases was validated by western blot 2 days after the transfection (n=2). The number of emergent clones was evaluated 9 days later (LS174T n=7, MCF7 n=7, Kolmogorov-Smirnov test, ***p<0.001). **C and D**. Analysis by RT-QPCR of E2F1 proliferative targets genes 3 days following the depletion of LARS or YARS with a single siRNA in senescent MCF7 **(C)** and senescent LS174T cells **(D)** (n=4 and 5 respectively, Kolmogorov-Smirnov test, * = p<0.05, ** = p<0.01).
