# Supplementary material for: tRNA biogenesis and specific aminoacyl-tRNA synthetases regulate senescence stability under the control of mTOR
Source: PLoS Genet. 2021 Dec 20;17(12):e1009953. doi: 10.1371/journal.pgen.1009953 (PMC8722728; doi:10.1371/journal.pgen.1009953)
Supplement: S9 Fig — (PDF) [file pgen.1009953.s009.pdf]

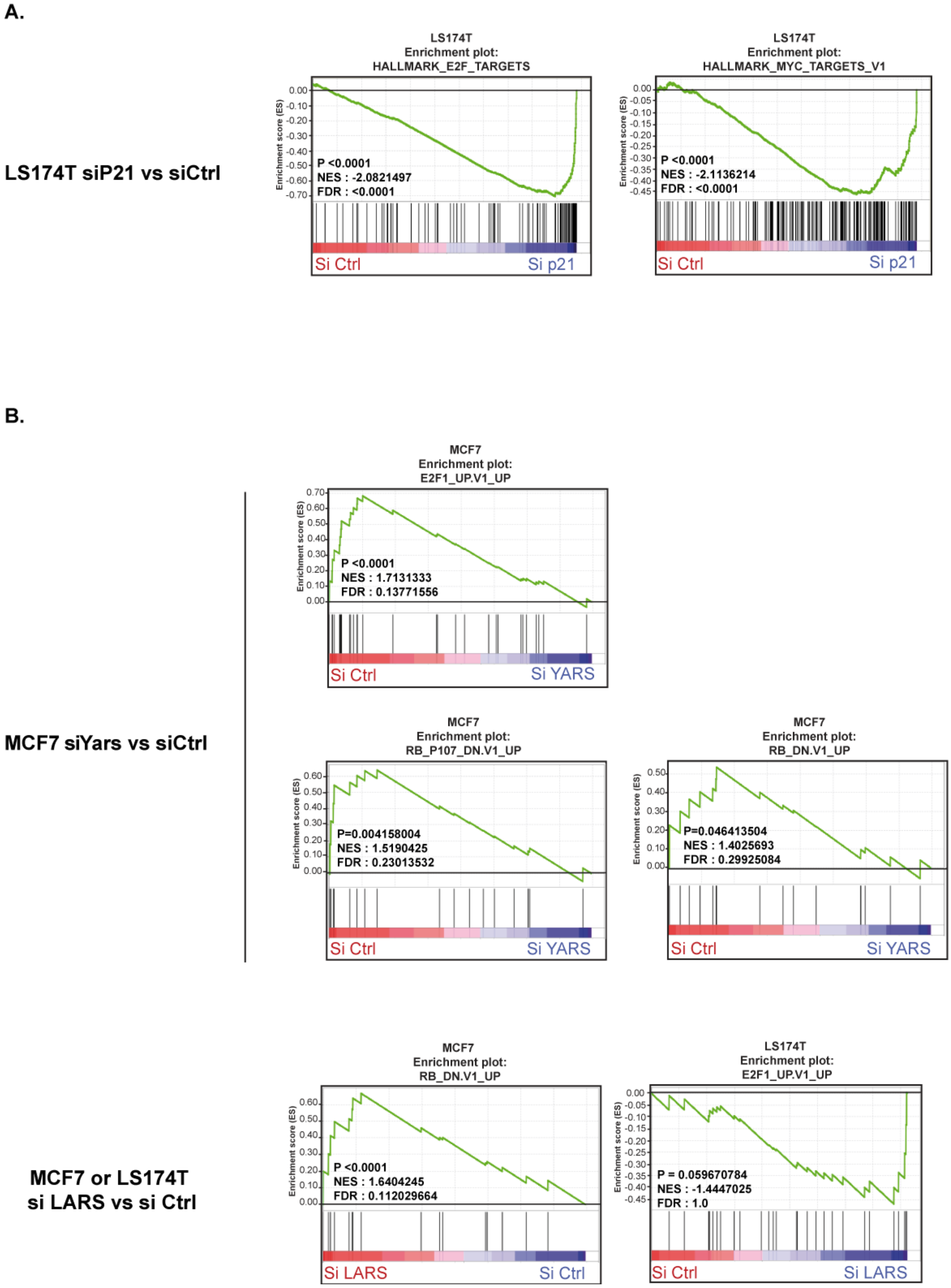

S9 Fig: Enrichment plot of the proteomic signatures.
Enrichment plot of Hallmarks or oncogenic Signatures obtained with mass spectrometry raw data analysis with GSEA software. **A.** These enrichment plot corresponds to the comparison of proteome of LS174T senescent cells 2 days after their transfection with a control siRNA or a smart pool of siRNA directed against p21 **B.** These enrichment plot corresponds to the comparison of proteome of MCF7 (left) or LS174T (right) senescent cells 3 days after their transfection with a control siRNA or a smart pool of siRNA directed against YARS or LARS.
