# Supplementary material for: tRNA biogenesis and specific aminoacyl-tRNA synthetases regulate senescence stability under the control of mTOR
Source: PLoS Genet. 2021 Dec 20;17(12):e1009953. doi: 10.1371/journal.pgen.1009953 (PMC8722728; doi:10.1371/journal.pgen.1009953)
Supplement: S10 Fig — (PDF) [file pgen.1009953.s010.pdf]

A.

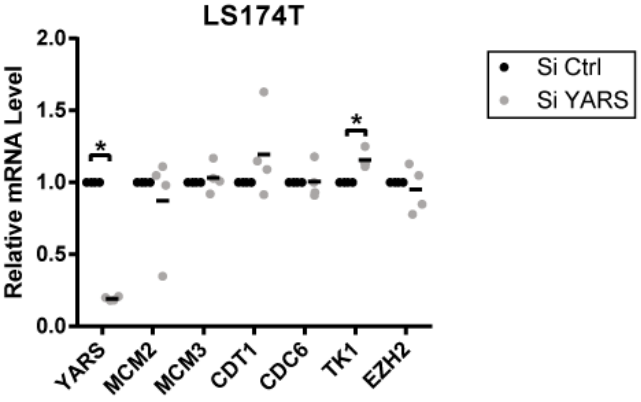

B.

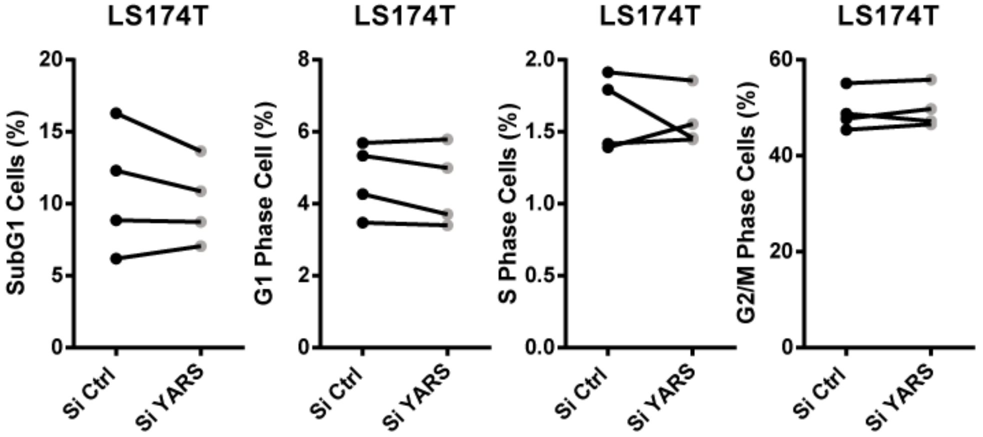

C.

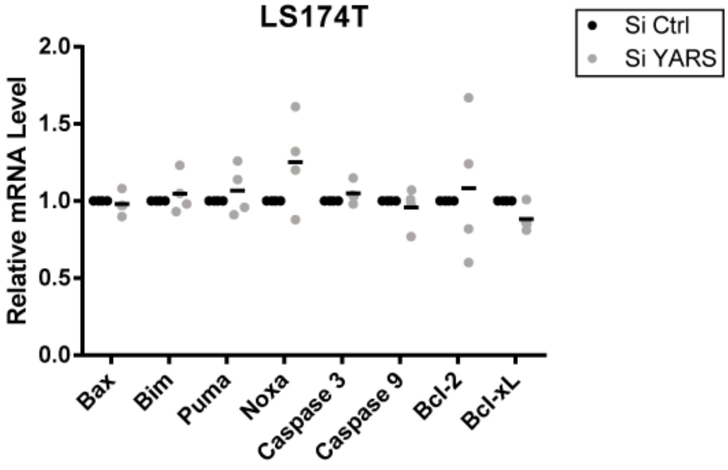

S10 Fig: Effect of YARS inhibition in LS174T colorectal cells.
A. LS174T senescent cells were transfected with a control siRNA or a smart pool of siRNA directed against YARS. Two days after the depletion, the expression of E2F1 proliferative targets was analyzed by RT-QPCR (n=4, Kolmogorov-Smirnov test,* = p<0.05). **B.** LS174T senescent cells were transfected with a control siRNA or directed against YARS. Two days after the depletion, FACS analysis was performed to analyze the cell cycle profile of the indicated cells (n=4).
C. LS174T senescent cells were transfected with a control siRNA control or a smart pool directed against YARS. Two days after the depletion, RT-QPCR was performed to analyse the expression of the indicated mRNAs (n=4).
