# Supplementary material for: tRNA biogenesis and specific aminoacyl-tRNA synthetases regulate senescence stability under the control of mTOR
Source: PLoS Genet. 2021 Dec 20;17(12):e1009953. doi: 10.1371/journal.pgen.1009953 (PMC8722728; doi:10.1371/journal.pgen.1009953)
Supplement: S1 Methods — (DOCX) [file pgen.1009953.s011.docx]

**Supplementary Methods**

**Chromatin immunoprecipitation (Chip)**

Cells were cross-linked with 1% formaldehyde (Sigma Aldrich) for 10 min at room temperature. Cross-linking was stopped by adding 0.125 mol/L glycine for 5 min. Cells were washed three times with cold phosphate-buffered saline (PBS). Cells were then scraped and washed three times with cold PBS. Pellets were resuspended in 1 mL of lysis buffer (5 mM PIPES, 85mM KCl, and 0.5% NP40). All buffers were supplemented with proteases and phosphatase inhibitors (1mM PMSF, 10 μg/ml aprotinin, 10 μg/ml leupeptin, 10 μg/ml pepstatin, 1 mM Na3VO4, and 50 mM NaF). Samples were incubated for 15 min at 4 °C and vortexed for 30 seconds every 2 minutes. Cells were centrifuged for 10 min, 16 000g at 4 °C. Supernatants were discarded and pellets were resuspended in 500 μL of sonicating buffer (10 mM EDTA, 1% SDS, and 50 mM Tris-EDTA, pH 8). Nuclear extracts were sonicated (20 cycles of 23 s of sonication and 25s on ice) to obtain DNA reverse cross-linked fragments with a size of 500-200 bases. Supernatants were diluted 10 times with IP buffer (0.01% SDS, 1.1%Triton X-100, 1.2 mM EDTA, 16.7 mM Tris-HCl (pH =8.0), and 167 mM NaCl). For each condition, 13 μg of chromatin were used (chromatin concentration was estimated by nanodrop on the reverse cross-linked chromatin). The chromatin was pre-cleared with 25 μl of beads for 2 hr on rotation at 4°C. Beads (Protein A/G Magnetic Beads, Thermofisher, 26162) were coated during the same time with 3 μg of the following antibodies : Rabbit Polyclonal POLR3A (Abcam, ab96328) or Rabbit IgG, polyclonal - Isotype Control (Abcam, ab171870). Each pre-cleared sample was then incubated with 45 μl of coated magnetic beads and DTT (1 mM) and BSA (10 μg/ml) added at the last moment. After overnight incubation at 4°C, the beads were washed successively with 1.5 ml of TSE1 Buffer (1%Triton X-100, 150 mM NaCl, 20 mM Tris-HCl, pH 8.1, 0.1% SDS, and 2 mM EDTA), 1.5mL of TSE2 buffer (1%Triton X-100, 500 mM NaCl, 20 mM Tris-HCl, pH 8.1,0.1% SDS, and 2 mM EDTA), and 1.5mL of TSE3 buffer 1% NP40, 1% sodium deoxycholate, 250mM LiCl, and 10mM Tris-HCl, pH 8.1). Following two washes in TE buffer (10 mM Tris-HCl and 1 mM EDTA), samples were eluted with 300 μL of fresh elution buffer (1% SDS and 0.1M NaHCO3). The cross-link was reversed by adding 24μL of NaCl (5 M) and 6μL of EDTA (0.5 M) to the samples and incubating overnight at 65 °C. DNA was purified using a High Pure PCR Template Preparation Kit (Roche) and analyzed by Q-PCR using 4 μl of the elution and 6 μl of Syber green containing the primers (5 μl of Syber green and 1 μl of primers (5 μM)).

**Mass spectrometry**

The technical approach was the same as previously reported [1].

*Creation of the spectral library*

In order to build the spectral library, peptide solutions of several protein samples were analyzed by a shotgun approach by micro-LC–MS/MS. Five pooled samples of breast, colorectal and blood tissues were prepared to obtain a good representation of the peptides. Each sample was fractionated by offgel fractionator in 24 fractions. Each fraction was separated into a micro-LC system Ekspert nLC400 (Eksigent, Dublin, CA, USA) using a ChromXP C18CL column (0.3 mm × 15 cm, 3 μm, 120 Å) (Eksigent) at a flow rate of 5 μL/min. Water and ACN, both containing 0.1% formic acid, were used as solvents A and B, respectively. The following gradient of solvent B was used: 0 to 5 min 5% B, 5 to 125 min 5% to 35% B, then 9 min at 95% B, and finally 9 min at 5% B for column equilibration. As the peptides eluted, they were directly injected into a hybrid quadrupole-TOF mass spectrometer Triple TOF 5600 + (Sciex, Redwood City, CA, USA) operated with a ‘top 30’ data-dependent acquisition system using positive ion mode. The acquisition mode consisted of a 250 ms survey MS scan from 400 to 1250 m/z, followed by an MS/MS scan from 200 to 1500 m/z (75 ms acquisition time, 350 mDa mass tolerance, rolling collision energy) of the top 30 precursor ions from the survey scan. The peptide and protein identifications were performed using Protein Pilot software (version 5.0, Sciex) with a human Swiss-Prot/TrEMBL concatenated target-reverse decoy database (downloaded in March 2016) containing 142,441 target human protein sequences, specifying MMTS as Cys alkylation. The false discovery rate (FDR) was set to 0.01 for both peptides and proteins. The MS/MS spectra of the identified peptides were then used to generate the spectral library for SWATH peak extraction using the add-in for PeakView Software (version 2.2, Sciex) MS/MSALL with SWATH Acquisition MicroApp (version 2.0, Sciex). Peptides with a confidence score above 99% as obtained from Protein Pilot database search were included in the spectral library.

*Relative quantification by SWATH acquisition*

LS174T cells were analyzed using a DIA method. Each sample (5 μg) was analyzed using the LC– MS equipment and LC gradient described above, using a SWATH-MS acquisition method. The method consisted of repeating the whole gradient cycle, which consisted of the acquisition of 35 TOF MS/MS scans of overlapping sequential precursor isolation windows (25 m/z isolation width, 1 m/z overlap, high sensitivity mode) covering the 400 to 1250 m/z mass range, with a previous MS scan for each cycle. The accumulation time was 50 ms for the MS scan (from 400 to 1250 m/z) and 100 ms for the product ion scan (230 to 1500 m/z), thus making a 3.5 s total cycle time.

*Data analysis*

The targeted data extraction of the SWATH runs was performed by PeakView using the MS/ MSALL with SWATH Acquisition MicroApp. PeakView processed the data using the spectral library created from the shotgun data. Up to ten peptides per protein and seven fragments per peptide were selected, based on signal intensity; any shared and modified peptides were excluded from the extraction. The retention times from the peptides that were selected for each protein were realigned in each run according to iRT peptides (Biognosys AG, Schlieren/Zürich, Switzerland) spiked in each sample and eluting along the whole time axis; the extracted ion chromatograms were generated for each selected fragment ion. PeakView computed a score and an FDR for each assigned peptide using chromatographic and spectra components; only peptides with an FDR of less than 5% were used for protein quantitation. The peak areas for peptides were obtained by summing the peak areas of the corresponding fragment ions; protein quantitation was calculated by summing the peak areas of the corresponding peptides. MarkerView (version 1.2, Sciex) was used for signal normalization, and differential abundance was tested by applying a T-test at the protein level.

The proteomics files are available via ProteomeXchange with identifier PXD029535

Submission details:

Project Name: tRNA Biogenesis and Specific Aminoacyl-tRNA Synthetases Regulate Senescence Stability Under the Control of mTOR

Project accession: PXD029535

**GSEA Analysis :**

Raw mass spectrometry data were loaded onto GSEA-4.1.0 software and the Hallmarks and Oncogene signatures were analyzed.

**Western Blot**

Following cell lysis with FASP Buffer (0.1 M Tris-HCL, 4% SDS, pH=7.6) containing a cocktail of inhibitors (10 μg/ml aprotinin, 10 μg/ml leupeptin, 10 μg/ml pepstatin, 1 mM Na3VO4, 50 mM NaF), lysates were sonicated and then boiled for 10 min. Proteins were separated on a SDS polyacrylamide gel and transferred to a PVDF membrane. Following a 1 hr incubation in 5% milk or 5% BSA, Tris-buffered saline (TBS), and 0.1% Tween 20, the membranes were incubated overnight at 4 °C with the following primary antibodies : p21waf1/cip1 (1/1000, Cell Signaling 2947), p-S6 ribosomal (S235/236) (1/1000, Cell Signaling, 2211), Chop (1/1000, Cell Signaling, 5554), HSC70 (1/1000, Santa Cruz, sc-7298), Bip (1/1000, Cell Signaling, 3177), p-Rb(S780) (1/1000, BD Pharmingen, 558385), Cyclin A (1/1000, Santa Cruz, sc-271682), TSC2 (1/1000, Cell Signaling, 4308), LARS (1/1000, Cell Signaling, 35509), TyrRS (1/1000, Santa-Cruz, sc-166741), CysRS (1/1000, Santa-Cruz, sc-390230), p-4E-BP1 (Thr37/46) (1/2000, Cell signaling, 2855), p-ULK1 (Ser757) (1/1000, Cell signaling, 14202), Raptor (24C12) (1/1000, Cell signaling, 2280), BRF1 (1/1000, Abcam, ab74221). Membranes were then washed three times with TBS with 0.1% Tween 20 and incubated for 45 minutes with the secondary antibodies listed below: Anti-rabbit IgG, HRP-linked antibody (1/3000, Cell Signaling, 7074), Anti-mouse IgG, HRP-linked Antibody (1/3000, Cell Signaling, 7076). Visualization was performed by chemiluminescence with a Fusion Solo (Vilber).

**RT-QPCR Analysis**

Total RNA was extracted with NucleoZOL (MACHEREY-NAGEL, Ref 740404.200) following the manufacturer's instructions with one exception: the step with RNAse-free water was repeated twice to reduce contamination by genomic DNA. Quantification of total RNA was performed by nanodrop.

Reverse transcription was performed after incubation of RNA with Random Primers (ThermoFisher Scientific, Ref 48190011) for 5 minutes at 70°C (critical step for tRNA expression analysis), then a mix containing M-MLV buffer, dNTPs, and M-MLV reverse transcriptase (ThermoFisher Scientific, Ref 28025013) was added to each tube and the samples were incubated for 1 hour at 37°C.

Quantitative PCR was performed using the Maxima SYBR Green/ROX qPCR master mix (ThermoFisher Scientific, Ref K0223).

Analysis was performed using the comparative CT method (2^(ΔCt)), according to the expression of three endogenous housekeeping gene TBP, PPIA and EEF1A1. All primers sequences are provided below.

**SA-β Galactosidase staining**

Cells were fixed for 10 min at room temperature in 2% formaldehyde, washed with PBS and incubated at 37°C in the absence of CO2 with freshly-made staining solution: 0.3 mg/mL of 5- bromo-4-chloro-3-indolyl-β-d-galactopyranoside)(X-Gal,Promega, V394A), 40 mM citric acid (Sigma), 40 mM sodium phosphate (Sigma) (stock solution (400 mM citric acid, 400 mM sodium phosphate) must be at pH 6), 5 mM potassium hexacyanoferrate (Sigma), 5 mM potassium ferricyanide (Sigma), 150 mM NaCl (Sigma), 150 mM MgCl2 (Sigma). SA-β galactosidase staining was observed after 16 hours.

**Flow Cytometry: CD47 extracellular staining:**

250 000 cells were incubated for 15 minutes in the dark at room temperature in 50µl of PBS-BSA 2% containing 200ng of APC mouse IgG1K isotype control (eBiosciences, 17-4714-42) or 200ng of APC antiCD47 (eBiosciences, 17-0479-42). The cells were then washed by adding 3ml of PBS-BSA 2% and centrifuged at 300g for 5 minutes. Cells were resuspended in 250µl of PBS and analyzed in the BD LSR II, 30 000 events were recorded per sample.

**MTT assay**

Cell viability and proliferation was evaluated by the MTT (3-4,5-dimethylthiazol 2,5- diphenyltetrazolium bromide) assay. Cells were seeded in 96-well clear-bottomed plates at a density of 2000 cells (LS174T) and 900 cells (MCF7) in RPMI medium containing 10% FBS. After 72 hr, 40 μL of 5 mg/ mL MTT solution was added to each well and incubated in a humidified 5% CO2 atmosphere at 37°C for 3 hours. After incubation, the cells were pelleted and dried. Next, 100μL of DMSO (dimethylsulfoxyde) were added to each well, and the cells were incubated at room temperature for 1 hr. Absorbance was measured by a microtiter plate reader at 562 nm (Tecan, Sunrise).

**Statistical analysis:**

In each graph, the bars correspond to the mean and the points represent the values obtained for the biological replicates. Differences were analyzed using a non-parametric test (Mann-Whitney or Kolmogorov-Smirnov for normalized data). * p<0.05, ** p<0.01 and *** p<0.001. When no stars are indicated on the graph it means that differences were not significant.

**References**

1. Guillon J, Petit C, Moreau M, Toutain B, Henry C, Roche H, et al. Regulation of senescence escape by TSP1 and CD47 following chemotherapy treatment. Cell Death Dis. 2019;10(3):199. Epub 2019/03/01. doi: 10.1038/s41419-019-1406-7. PubMed PMID: 30814491; PubMed Central PMCID: PMCPMC6393582.
